# A comprehensive review of computational cell cycle models in guiding cancer treatment strategies

**DOI:** 10.1038/s41540-024-00397-7

**Published:** 2024-07-05

**Authors:** Chenhui Ma, Evren Gurkan-Cavusoglu

**Affiliations:** Department of Electrical, Computer and Systems Engineering, Case Western Reserve University, Cleveland, OH USA

**Keywords:** Software, Computational science

## Abstract

This article reviews the current knowledge and recent advancements in computational modeling of the cell cycle. It offers a comparative analysis of various modeling paradigms, highlighting their unique strengths, limitations, and applications. Specifically, the article compares deterministic and stochastic models, single-cell versus population models, and mechanistic versus abstract models. This detailed analysis helps determine the most suitable modeling framework for various research needs. Additionally, the discussion extends to the utilization of these computational models to illuminate cell cycle dynamics, with a particular focus on cell cycle variability, crosstalk with signaling pathways, tumor microenvironment, DNA replication, and repair mechanisms, underscoring their critical roles in tumor progression and the optimization of cancer therapies. By applying these models to crucial aspects of cancer therapy planning for better outcomes, including drug efficacy quantification, drug discovery, drug resistance analysis, and dose optimization, the review highlights the significant potential of computational insights in enhancing the precision and effectiveness of cancer treatments. This emphasis on the intricate relationship between computational modeling and therapeutic strategy development underscores the pivotal role of advanced modeling techniques in navigating the complexities of cell cycle dynamics and their implications for cancer therapy.

## Introduction

The eukaryotic cell cycle, a cornerstone of cellular vitality, is an ordered and tightly regulated sequence divided into four primary phases: G1, S, G2, and M. This sequence supports critical processes such as organelle reorganization and chromatin licensing during the G1 phase, DNA replication in the S phase, protein synthesis during the G2 phase, and chromosomal segregation culminating in cytokinesis, which produces two daughter cells during the M phase^1^. The cell cycle is integral to maintaining homeostasis, influencing processes such as cell death^2^, metabolism^3^, cell motility^4^, cell migration^5^, nutrient uptake^6^, and DNA repair.

Cell cycle progression is tightly controlled in normal tissues but aberrant during disease progression, such as in tumorigenesis. Normal cellular functions rely on the precise regulation of the cell cycle, supported by mechanisms spanning from transcriptional regulation^7^ to post-translational modifications. Cell cycle regulators, including cyclin-dependent kinases (CDKs), cyclins, p53, and cyclin-dependent kinase inhibitors (CKIs), balance the processes of cell growth and division with the cell needs to differentiate or undergo programmed cell death^8^, by integrating signals from diverse extracellular and intracellular sources^9^. This cooperation is safeguarded by the checkpoint control and DNA damage response pathway, which would halt cell cycle progression for repair upon DNA damage caused by exogenous and endogenous factors in any cell cycle phase, ensuring genetic integrity or inducing apoptosis in severe cases^10^. Genomic stability is maintained by the high fidelity of DNA replication and the accurate distribution of chromosomes among daughter cells during mitosis^11^. The decision for daughter cells to re-enter the cell cycle or enter a state of quiescence hinges on various factors. These factors include internal aspects such as the mother cells’ DNA integrity^12^, cell type^13^, and differentiation status^14^, as well as external cues such as nutrient availability and signals from neighboring cells^15^. This complex decision-making process leads to observed heterogeneity in the quiescence-to-proliferation transition, attributable to deterministic memory effects from the preceding cell cycle and the inherent randomness introduced by the stochastic dynamics of the RB-E2F bistable switch^16^.

In cancer, a critical disruption occurs within the cell cycle, driving uncontrolled cellular proliferation^17^. This dysregulation stems from the accumulation of genetic mutations that undermine cell cycle checkpoints and impair apoptotic responses^18,19^. Such alterations sustain proliferative signaling and impair growth suppressor pathways, leading to unchecked cellular division, even without normal growth stimuli^20^. Such genetic instability often coincides with the emergence of cells with self-renewal capacities^21^. This review focuses on the dysregulation of the cell cycle within the context of cancer. We aim to explore this phenomenon not just as a hallmark of cancer but also as a promising target for therapeutic interventions.

Mathematical modeling is a powerful tool that transforms descriptive knowledge into quantitative insights, enabling scientists to navigate the intricacies of biological systems characterized by networks of interacting variables. These models serve as virtual laboratories, allowing for hypothesis testing and therapeutic exploration without the constraints of traditional experimentation. Another key strength of mathematical models is their capacity to decode non-intuitive experimental outcomes and to explore the impact of variables. Furthermore, these models’ predictive power enables the prediction of a spectrum of conditions, such as cellular responses to drugs, environmental shifts, or genetic alterations.

**Fig. 1:**
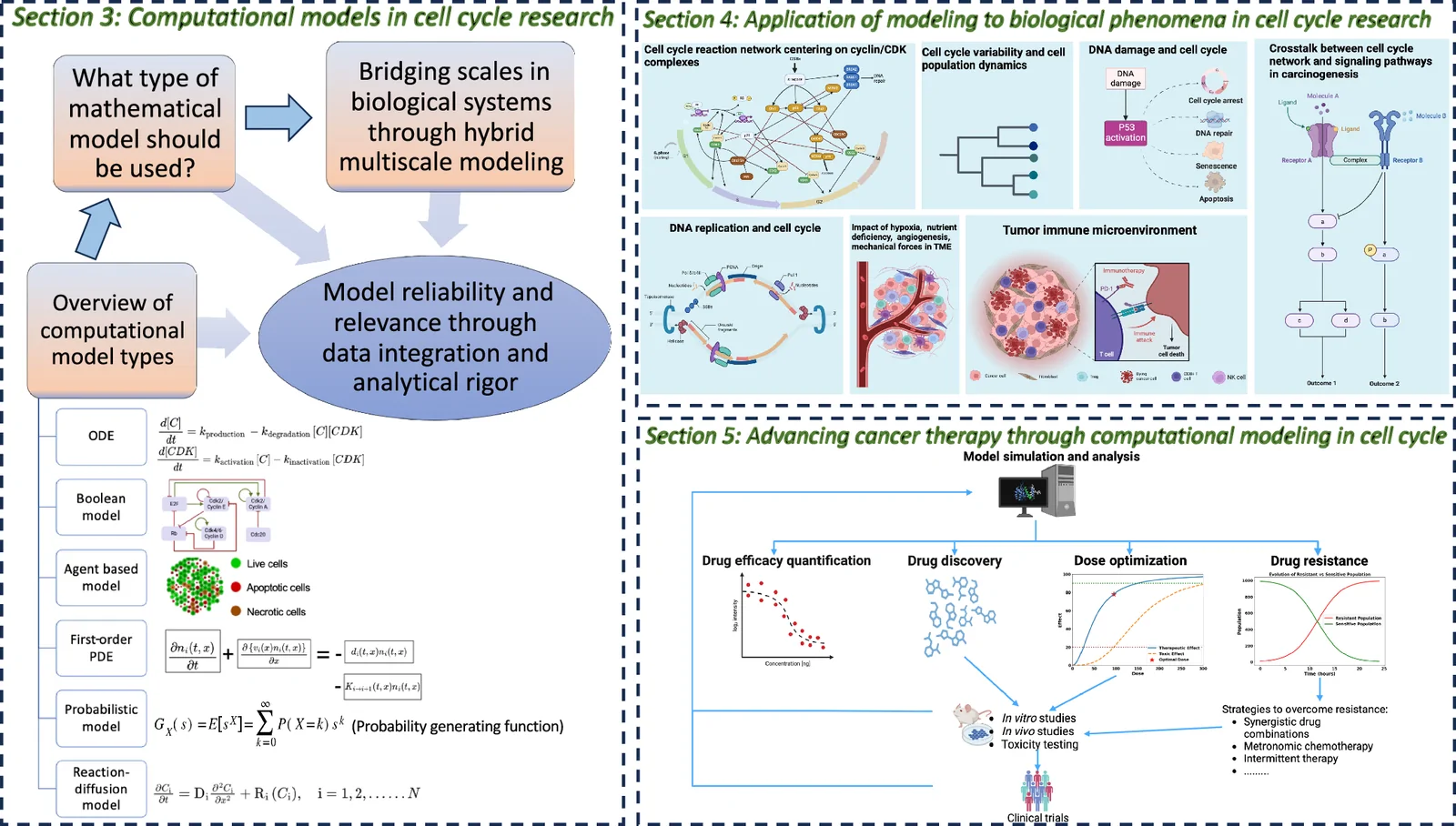
Overview of computational modeling in cancer cell cycle research. Panels for sections ''Applications of cell cycle models to model biological phenomena'' and ''Enhancing cancer treatment strategies through computational modeling of the cell cycle'' were created with BioRender.com.

**Fig. 2:**
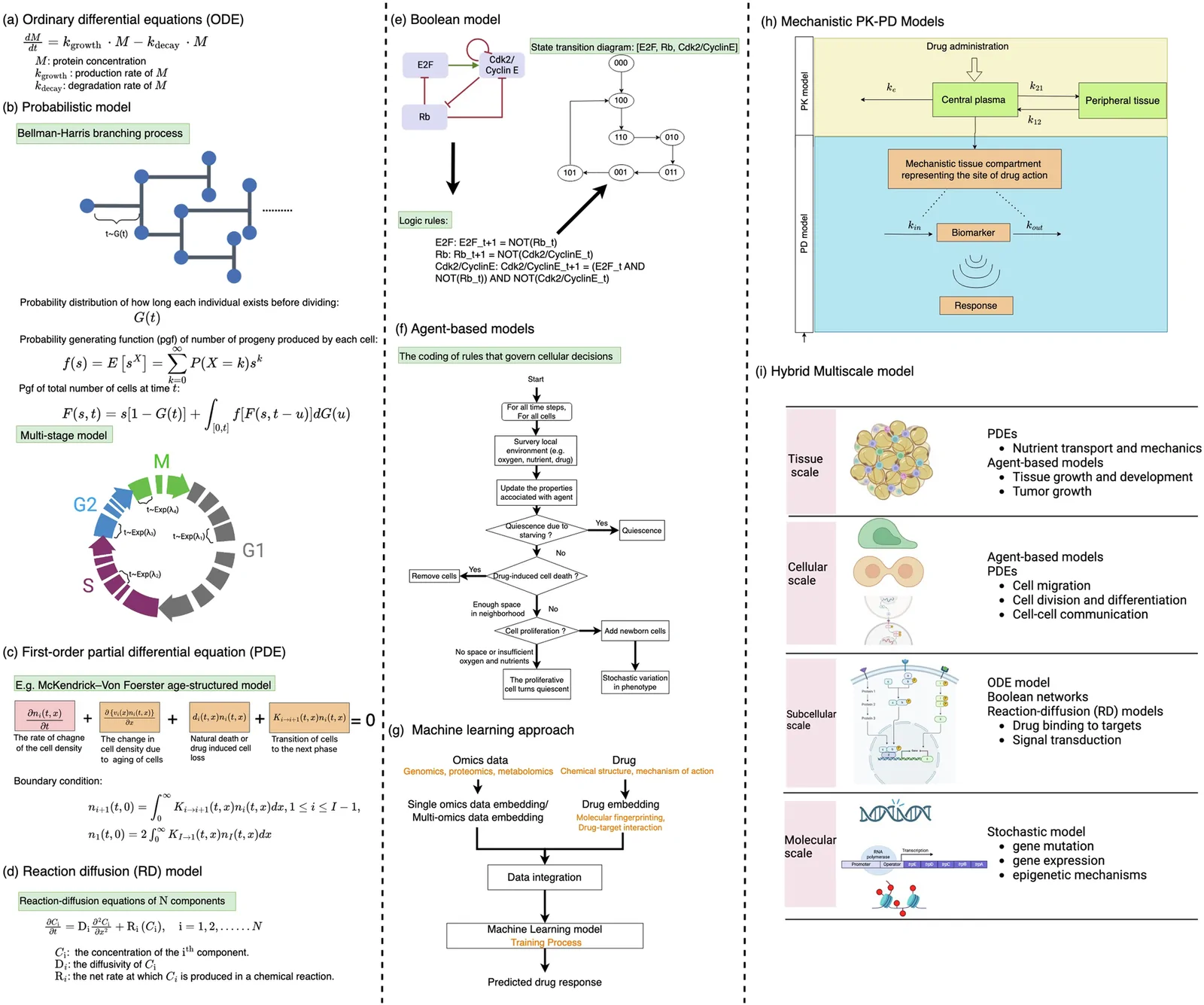
Overview of computational modeling techniques. This figure depicts various mathematical frameworks used in the study of cellular processes. **a** Ordinary differential equations capture dynamic changes in molecular concentrations over time. **b** Probabilistic models, including the Bellman-Harris branching process and multi-stage models, explore cell division probabilities and lifespans. **c** First-order partial differential equations model cell population dynamics with age structures. **d** Reaction-diffusion models address the spatial distribution of cellular components. **e** Boolean models simplify complex regulatory networks into binary interactions. **f** Agent-based models illustrate individual cell decisions within their microenvironments. **g** Machine learning leverages omics data and drug structure data to predict drug responses. **h** Mechanistic PK-PD models link pharmacokinetics and pharmacodynamics for drug action insights. **i** Hybrid multiscale models bridge molecular details with tissue-level phenomena. Panels **b** and **i** were created with BioRender.com.

This review article aims to be a comprehensive resource for researchers looking to identify the appropriate modeling paradigm to incorporate in their work that focuses on studying the cell cycle computationally (Fig. 1). We first provide the biological foundation of the cell cycle in ''The biological foundation of the cell cycle'', discussing the key mechanisms and aberrations in the cell cycle leading to carcinogenesis. We then explore the tumor microenvironment (TME), a complex network of extracellular matrix (ECM), diverse cell types, and vascular structures, underscoring its dynamic interactions and critical influence on cancer progression and therapeutic response. In Section ''Computational models in cell cycle research'', we review computational models that have been proposed to quantify cell cycle dynamics and demonstrate their capacities to study biological phenomena at different scales. The frameworks and the underlying mathematics of these models are shown in Fig. 2. Then, we present their unique strengths and limitations, providing guidance on how to choose the appropriate model for the specific research problem. In addition, recognizing that these models complement rather than compete with each other, we demonstrate their synergistic potential and joint coordination in the pursuit of comprehensive understanding. Addressing the complexity of cellular processes requires robust analytical and modeling approaches. Therefore, in Section ''Computational models in cell cycle research'', we review the techniques applied in parameter estimation and sensitivity analysis, which are essential for ensuring model robustness. Integrating the biological details into the models and linking them to empirical data ensures their utility, which is also discussed in Section ''Computational models in cell cycle research''.

In Section ''Applications of cell cycle models to model biological phenomena'', we review how computational models lead to insights into the regulation, variability, and interaction of the cell cycle with cellular processes. We demonstrate how these models provide a detailed understanding of the cyclin/CDK networks that drive cell cycle progression and the essential coordination of DNA replication and repair critical for maintaining genomic integrity. Our discussion underscores the pivotal role of computational models in revealing how variability in cell cycle durations affected by factors such as cell size influences population dynamics. These models expose how control mechanisms introduce significant variabilities to critical aspects of the cell cycle, including growth and division, and delineate how stem cell behavior impacts tissue homeostasis. Additionally, we explore how computational approaches help to illustrate the intricate interplay between cell cycle regulation and signaling pathways, shedding light on the underlying mechanisms that drive carcinogenesis. Moreover, the section details how computational modeling is crucial in understanding the tumor microenvironment’s influence on cell cycle dynamics and tumor evolution, encompassing factors such as hypoxia, nutrient scarcity, angiogenesis, and mechanical forces. We also highlight how mathematical models, particularly agent-based models, are utilized to assess the role of the tumor immune microenvironment in influencing the outcomes of immunotherapy.

Finally, we review the computational approaches for optimizing cancer treatment targeting cell cycle in Section ''Enhancing cancer treatment strategies through computational modeling of the cell cycle''. The development of effective cancer treatment is thwarted by tumor heterogeneity, lack of predictive biomarkers, the occurrence of drug resistance, and limitations inherent in preclinical models. Utilizing mathematical modeling, coupled with advancements in parallel computing technologies and access to large-scale supercomputing resources, has the potential to significantly accelerate scientific breakthroughs, refine design methodologies, and enable more precise strategic decisions. We present computational approaches that have been employed to study the effect of cell cycle-dependent anticancer treatment on the proliferation dynamics of the cancer cell population, strategies for overcoming emergent drug resistance, and how these insights contribute to the discovery and optimization of therapeutic interventions. The list of abbreviations and their full names used in the article can be found in Table 1.

**Table 1:** List of abbreviations and their full names used in the article

Abbreviation	Full Name
ABMs	Agent-based models
ANN	Artificial neural network
APC	Antigen-presenting cell
BrCa	Breast cancer
CDKs	Cyclin-dependent kinases
CFSE	Carboxyfluorescein succinimidyl ester
CKIs	Cyclin-dependent kinase inhibitors
DCIS	Ductal carcinoma in situ
DDR	DNA damage response
DRL	Deep reinforcement learning
DSBs	Double-strand breaks
ECM	Extracellular matrix
EMT	Epithelial-mesenchymal transition
FUCCI	Fluorescent ubiquitination-based cell cycle indicator
HSC	Hematopoietic stem cell
LET	Linear energy transfer
mAbs	Monoclonal antibodies
MAPK	Mitogen-activated protein kinase
MCMC	Markov chain Monte Carlo
MIDD	Model-informed drug development
ML	Machine learning
MSMs	Multi-stage models
MTD	Maximum tolerated dose
NSCLC	Non-small cell lung cancer
OCT	Optical coherence tomography
ODEs	Ordinary differential equations
OvCa	Ovarian cancer
PaCa	Pancreatic cancer
PDEs	Partial differential equations
PPI	Protein-protein interaction
PSPMs	Physiologically structured population models
QSP	Quantitative systems pharmacology
RBF	Radial basis function
RD	Reaction-diffusion
RMSE	Root mean square error
RPIM	Radial point interpolation method
SDEs	Stochastic differential equations
SMI	Small-molecule inhibitor
SPN	Stochastic Petri net
spQSP	Spatial quantitative systems pharmacology
SSA	Stochastic simulation algorithm
SVM	Support vector machine
TGI	Tumor growth inhibition
TME	Tumor microenvironment
VEGF	Vascular endothelial growth factor
VP	Virtual patient
WCMs	Whole-cell models

## The biological foundation of the cell cycle

### Key mechanisms of the normal cell cycle

**Fig. 3:**
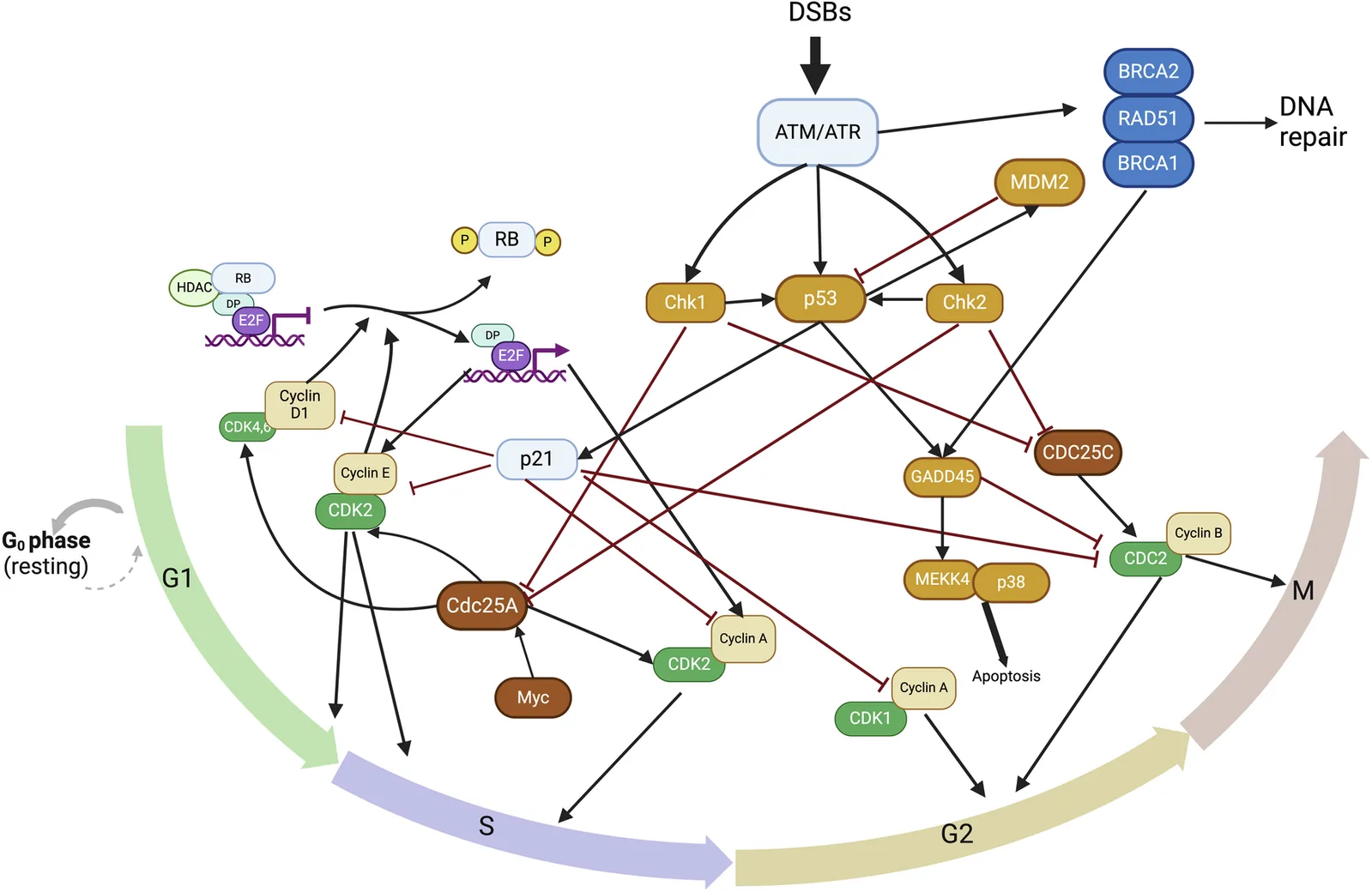
Cell cycle regulation and DNA damage response pathways. The diagram illustrates the network of interactions governing the cell cycle, from the quiescent G0 phase to the G1, S, G2, and M phases. Key proteins and complexes, such as cyclins, CDKs, p53, and ATM/ATR, are central to directing cell cycle progression and responding to DNA insults, indicated by double-strand breaks (DSBs). These intertwined pathways safeguard proper DNA replication, repair, and the cell’s responses to external stressors, with possible outcomes spanning from cell cycle arrest to apoptosis. Created with BioRender.com.

The cell cycle, often referred to as “the cell cycle oscillator” due to its cyclical and rhythmic progression, progresses through the sequenced phases of G1, S, G2, and M^22,23^. This progression is propelled by the transient activation of CDKs as depicted in Fig. 3. Checkpoints within the cell cycle ensure genomic integrity. The G1 restriction point, intricately regulated by the RB pathway, dictates commitment to DNA synthesis or entry into quiescence in continuously cycling cells^24^. S phase checkpoint, activated by the ATR/Chk1 pathway, monitors DNA replication and repair, stabilizing replication forks, coordinating repair, and inducing a G2/M cell cycle arrest^25^. The G2/M checkpoint, reliant on ATM, ATR, Chk1, and Chk2, monitors DNA replication completion and the presence of damage before mitosis initiation^26^. Additionally, the spindle assembly checkpoint, involving MAD2, BUBR1, and CDC20, ensures the chromosomes’ proper alignment on the mitotic spindle. The network also manages transitions between cell cycles and mitotic exits and maintains stability against inherent fluctuations and external disturbances. Some stem cells, including embryonic stem cells and pluripotent stem cells, are thought to lack a restriction point, allowing for rapid proliferation^27^.

### Aberrations in the cell cycle leading to cancer

Tumors behave as complex, self-organizing, opportunistic, and dynamic systems. Cancer progression is essentially an evolutionary process, and transitioning from a mere tumor to full-fledged malignancy necessitates that these cells navigate and adapt to various microenvironmental proliferation barriers. These include hypoxia, acidosis, ischemia, and limited availability of space and resources^21,28,29^. Genomic destabilization is an early event in carcinogenesis, setting the stage for cancer progression. This instability manifests on various levels, from DNA sequence changes to chromosomal abnormalities^30,31^. As carcinogenesis progresses, genotypical and phenotypical changes (e.g., epithelial-mesenchymal transition (EMT), cancer stem-like properties, metabolic reprogramming^32^) help cells adapt to hostile environments^28^. Gain-of-function mutations of proto-oncogenes (e.g., ERBB2, P13K, Ras GTPase, and the c-Myc) and loss-of-function mutations in tumor suppressor genes (e.g., TP53, BRCA1/2, PTEN)^33,34^ result in defective checkpoint function, causing the cell cycle to misregulate. Instead of delaying or arresting cell cycle progression in response to DNA damage, these defective pathways cause damaged DNA to replicate. This replication, in turn, amplifies genetic modifications and disrupts apoptosis pathways^35,36^. Additionally, abnormal activation of signal transduction pathways (Wnt, Notch, IGF, PI3K/Akt, NF-*κ*B, Hh) contributes to increased proliferation^37^. Particularly notable is the metabolic shift toward aerobic glycolysis, known as the Warburg effect, in hypoxic regions^21^. These molecular and metabolic changes are tightly linked to the cell cycle, guiding each stage of cancer progression, from invasion to dissemination^38^. Cells evolve the capacity for uncontrolled growth and division irrespective of external mitogens, gaining the ability to escape both apoptosis and differentiation^39^.

Loss of control over the cell cycle regulatory system is a common occurrence in cancer^36^. During tumorigenesis, dysregulation of the cell cycle is the combined result of various factors, including recurrent alterations and epigenetic changes in genes that directly regulate the cell cycle, influences of environmental stressors, and alterations in signaling pathways that are not primarily associated with cell cycle regulation but can modulate cell cycle regulators (such as mitogen-activated protein kinase (MAPK), Ras signaling pathways, and Pl3K/Akt/mTOR^40^). Dysregulation of the cell cycle is linked to carcinogenesis for several reasons. First, cell cycle dysregulation is a pivotal contributor to genetic instability. This instability arises from mutations or alterations in the components of pathways responsible for DNA damage response and repair mechanisms^41–43^ and due to checkpoint deficiencies^44,45^. Second, it can promote the emergence of cell phenotypes with proliferative advantage. This aberration appears as uncontrolled cell proliferation, resistance to cell death, and dysregulation that renders the cells less responsive to typical physiological controls, such as those imposed by limited nutrient availability or hypoxic conditions. Third, the dysregulated cell cycle can lead to changes in the TME, including increased secretion of growth factors and cytokines that promote tumor growth, invasion, and metastasis. This dysregulation is accompanied by a metabolic shift toward aerobic glycolysis to satisfy the heightened energy demands of proliferating cells, resulting in elevated lactate production. Consequently, this increases the acidity within the TME, particularly in its hypoxic core, which in turn can stimulate angiogenesis and contribute to an immune-suppressive environment^46,47^.

### The tumor microenvironment (TME): a critical factor in cancer progression and therapy resistance

TME consists of an extracellular matrix (ECM), multiple types of stromal cells (such as fibroblasts, mesenchymal stromal cells, pericytes, and adipocytes), immune cells (including T and B lymphocytes, natural killer cells, and tumor-associated macrophages), and an extensive network of blood and lymphatic vessels. The reciprocal interactions between tumor cells and TME components influence every stage of carcinogenesis^48^. TME dynamically changes and remodels in parallel with tumor cell proliferation governed by dysregulated cell cycle mechanisms, thereby contributing to the heterogeneity of tumor cells characterized by varied genetic and phenotypic traits^49,50^.

The ECM, beyond providing structural support, actively participates in signaling processes that regulate cancer cell behavior, migration, and invasion^51^. This role is further enhanced by the biomechanical properties of the ECM, such as stiffness, which influences cancer cell behavior^52^. Concurrently, both the innate and adaptive immune systems play a fundamental role in counteracting tumor growth through mechanisms like apoptosis induction and cell cycle arrest^53^. However, when evolutionary pressures skew this balance toward tumors that have developed strategies for immune evasion or immunoediting, the risk of malignancy increases. Within this complex ecosystem, TME plays a pivotal role in immune suppression and evasion, enabling tumor cells to escape immune-mediated destruction through mechanisms such as the recruitment of immunosuppressive cells (such as regulatory T-cells and myeloid-derived suppressor cells) and the expression of immune checkpoint molecules^54^. Hypoxia within the TME acts as a critical catalyst for this cellular reprogramming, simultaneously promoting the secretion of factors such as vascular endothelial growth factor (VEGF). This secretion leads to angiogenesis and lymphangiogenesis, processes that are crucial as they provide the growing tumor with additional nutrients and oxygen, essential for tumor cells’ survival and expansion. These newly formed vascular networks not only facilitate the metastatic spread of tumor cells but also contribute to the genetic and phenotypic heterogeneity observed within cancer populations, thus complicating treatment strategies and outcomes^55^.

These intricate interactions also significantly contribute to therapeutic resistance, as the TME can create physical barriers to drug delivery, secrete soluble factors that neutralize therapeutic agents, and induce drug resistance mechanisms. Recent advances in our understanding of the TME, fueled by technologies such as spatial transcriptomics and advanced imaging, have unveiled cellular heterogeneity and spatial organization within it^56^. Building on these insights, potential strategies for disrupting TME-regulated cancer cell growth include targeting stromal cells to reverse their pro-tumorigenic functions^57^ and reprogramming the immune microenvironment to restore anti-tumor immunity^58^.

## Computational models in cell cycle research

### Ordinary differential equation models (ODEs)

ODEs that are commonly employed for modeling signaling pathways offer a straightforward way to convert comprehensive reaction networks into mathematical representations^59^. This method is particularly effective in studying cell cycle kinetics, as it can accurately describe the timing and sequence of events that regulate cell division^60,61^. By modeling the underlying chemical reactions of the cell cycle, ODEs provide a platform from which researchers can derive clear, quantitative predictions of dynamic behaviors. They do this by describing the kinetics of biochemical reactions through well-established principles such as the law of mass action, Michaelis-Menten kinetics, or Hill functions. This mathematical framework allows for the precise description of how concentrations of different cellular components change over time, offering insights into how alterations in signaling pathways can influence cell cycle progression and how these changes can adapt to or be impacted by disruptions, such as those caused by genetic mutations or external stimuli. To derive these insights, modelers frequently employ sensitivity analysis and in silico predictions using ODE models. Sensitivity analysis involves systematically varying model parameters, such as rate constants for CDK activation or synthesis rates of cyclins, to understand their impact on outcomes such as cell cycle timing and phenotypes^62^. Bifurcation analysis is crucial for examining how small variations in control parameters, key variables that influence system dynamics, can cause qualitative changes, such as transitions between different steady states in cell cycles. This analysis helps identify critical thresholds where these shifts occur^63–67^. Time scale analysis differentiates between excitation and relaxation periods in the cell cycle by examining the Jacobian’s eigenvalues^68^. Negative eigenvalues during relaxation periods indicate the system is moving toward a stable state. In contrast, a positive eigenvalue in excitation periods signals instability, marking important cellular transitions. The use of in silico predictions involves running the ODE model under various hypothetical conditions to see how changes in the cellular environment or genetic mutations might affect the cell cycle^69,70^. These model predictions are then compared with experimental data, serving either to validate the model or to generate new hypotheses for experimental testing.

Nonetheless, ODE modeling faces several challenges. The primary one is the precise determination of the kinetic parameter values. While these parameters should ideally be derived from experimental data, in many cases, they are not readily available. This lack of data necessitates reliance on estimation techniques, and modelers frequently resort to calibrating these values to align with expected behaviors. Model calibration enables the practical application of ODE models in scenarios where not all parameters can be experimentally determined. To enhance the predictive accuracy of these models, there is an emphasis on selecting appropriate calibration techniques that strive to derive kinetic parameters with certainty and identifiability, particularly when limited time-course data are available^71–73^. Another challenge is accounting for cell population heterogeneity. ODE models are typically well-suited for scenarios where spatial factors, such as molecular diffusion and cellular localization, do not significantly influence the simulation outcomes or can be considered uniform. This simplification, though sometimes necessary, can lead to the omission of important spatial dynamics within the cellular environment, potentially compromising the model’s fidelity to real biological behaviors^74^. Moreover, ODEs are inherently deterministic and thus represent the mean behavior of populations, which overlooks the variability inherent in individual cells. To address this, ODE models can be converted into stochastic chemical kinetic models using a master equation by leveraging conversion strategies outlined in the literature^75^. This equation governs the probabilities of the quantities of each molecular species using parameters from the original ODE model. Exact simulations of these stochastic models can be carried out using methods such as the stochastic simulation algorithm (SSA) and the *τ*-leap approximation. To ensure the validity of SSA when applied to mechanistic models with complex order kinetics, it is essential to employ appropriate model reduction techniques. Stochastic differential equations (SDEs) offer another approach by incorporating randomness into the differential equations that describe the evolution of molecular species concentrations^76^. Despite these complexities, ODEs continue to be a fundamental tool in computational biology for their ability to provide a comprehensive framework for understanding complex biochemical networks within a manageable computational cost.

### Probabilistic models

Probabilistic models are fundamentally grounded in the principles of probability theory, which mathematically describes the randomness and uncertainty inherent in complex biological systems^77^. These models employ stochastic methods to account for the inherent randomness of events within cellular processes, such as gene expression, protein synthesis, and cell cycle dynamics. By applying probability distributions and stochastic equations, these models can precisely simulate and predict the range of possible behaviors and outcomes within a population of cells. This approach is crucial for analyzing variations in cell division times influenced by molecular and environmental interactions, thus enabling researchers to understand and predict the probabilistic nature of cellular behavior and its impact on population dynamics.

However, probabilistic models come with certain limitations. The requirement for substantial data to accurately define probability distributions is a major hurdle in biological settings, and the models’ accuracy heavily depends on the plausibility of their underlying assumptions, where inaccuracies can lead to significant discrepancies in predictions^78^. Moreover, as the complexity of biological systems increases, the scalability of probabilistic models becomes problematic. The computational demands for processing large networks and datasets can grow exponentially, making it challenging to execute these models on a large scale.

Using probabilistic models like branching processes and multi-stage models (MSMs) enables the investigation of how variations in division times affect cell population dynamics, thus illuminating stochastic elements of cellular behavior. The branching process demonstrates the dynamics of proliferating cell populations transitioning through generations. It is primarily applied to study the transient stage leading to the asymptotic log phase where populations maintain balanced growth with constant cell cycle phase percentages. The model begins with a single ancestor at time zero, which lives for a certain period before independently producing a random number of progenies based on a probability distribution, each initiating its own branching process. There are two types of branching processes: the classic branching process, where each cell has a fixed lifespan, and the Bellman-Harris process, allowing random and unique lifespans for each cell^79^. This variability in cell lifespan makes the Bellman-Harris process a more realistic model for studying cell division. Central to the Bellman-Harris process is the concept of generation expansion inherent in the renewal theory. This theory analyzes events that occur randomly in time, focusing on the distribution of intervals between successive events. On the other hand, MSM partitions the cell cycle into multiple exponentially distributed sequential stages leading to division^80–83^. This method approximates the probability density of the time until division by convolution of these exponential distributions, forming an Erlang distribution^84^. The optimal number of cell cycle stages can be determined from the best fit of an Erlang distribution to the experimental cell cycle duration^80^. It retains the mathematical advantages because of its closed-form probability density function and memoryless property^80^.

The foundational difference between these models lies in their mathematical basis: branching processes use renewal equations for event recurrences, whereas MSM, through partitioning, leverages the Markov process’s memoryless nature. In MSM, each stage of the cell cycle is modeled as an exponential phase with a certain rate, and transitions between stages are independent of the time already spent in the current stage. This memorylessness is a key characteristic of the Markov process. Moreover, because each transition depends only on the current state and not on how it was reached, MSMs enable the efficient simulation of cell cycle dynamics using the Gillespie algorithm^80,85^. In fact, research such as that by Chao et al. verifies the statistical independence between the cell cycle phases by examining the effects of perturbing phase durations through oncogene activation, inhibition of DNA synthesis, reduced temperature, and DNA damage. Their findings indicate that despite significant variations in durations across cell populations, phase durations remained statistically uncoupled in individual cells, thus validating the MSM’s foundational assumption of phase independence^86^.

### First-order partial differential equations (PDEs)

PDEs model multidimensional changes, describing the variation of a function with respect to several variables. A classic example of PDEs applied in the biological context is the McKendrick-Von Foerster model for the cell cycle, which is particularly renowned for its application to cell population dynamics.

The McKendrick-Von Foerster model employs age-structured dynamics through a system of nonlinear PDEs to describe the densities *n*_*i*_(*t*, *τ*) of cells in phase *i*, of age *τ*, at time *t*. The model accounts for the changes in population density over time, considering both the age of the individuals and their progression through different phases of a biological process, such as the cell cycle. Initial conditions define the starting cell phase distribution at time 0, while boundary conditions specify the age-zero cell distribution at any time. For cell cycle analysis, it uses periodic functions or transition age probability distributions to define transition rates between phases, highlighting how cell population variability stems from age differences of cells during the division cycle^87–94^.

McKendrick-Von Foerster’s model is classified within the realm of physiologically structured population models (PSPMs) due to its use of age as the primary structuring variable to describe population dynamics. In PSPMs tailored to cell cycle dynamics, structural variables can also correspond to a variety of cellular properties, including volume^95^, size^96,97^, DNA content^95^, cell culture time, bromodeoxyuridine (BrdUrd) uptake^98^, spatial position^97^, or a combination of these properties^95,97,99^. These structural variables are mathematically defined as internal coordinates in the individual state space or admissible individual states (i-states)^100^, allowing for the modeling of cells’ maturation on the physiological level. The integration of structure variables brings the model closer to the biological reality, overcoming the limits of simpler, phenomenological equations (such as exponential growth, logistic functions, etc.) often adopted to characterize cell population growth profiles.

PDE models come with inherent challenges. The models are governed by PDEs that can be challenging to solve analytically, especially in irregular domains or when the system involves complex boundary conditions. This complexity often necessitates sophisticated numerical methods, such as the method of characteristics^101,102^, finite difference method, meshfree methods like Radial Point Interpolation (RPIM)^103^ and Radial Basis Function (RBF)^104^. Another challenge lies in parameter calibration. There is growing interest in developing efficient estimation methods for PDEs^105,106^.

Reaction-diffusion (RD) models RD equations are instrumental in elucidating self-regulated pattern formation of key substances within extracellular matrices and intracellular environments, where molecules are organized into signaling pathways^107^. These equations model the spatiotemporal distribution of substances by incorporating diffusion processes that are microscopically driven by random molecular movements and macroscopically result in a net flux proportional to the concentration gradients. The reaction terms in RD models accurately describe the chemical or biological transformations that these substances undergo. This comprehensive modeling allows for detailed studies of the physical movement and interactions of substances across cellular compartments. The inherent need for conserving energy in transporting small cellular components underscores the suitability of RD models, as diffusion operates without an energetic cost, provided concentration gradients are maintained. RD models find extensive application in various cellular functions in eukaryotic cells, including cell division, chemotaxis, signaling cascades, and cell motility^108^. RD can also be applied to study the spatial diffusion of cells, an easily overlooked yet essential factor in determining the collective behavior of the cell population. For example, Adimy et al. applied RD models to studying the dynamics of hematopoietic stem cell (HSC) populations during the cell cycle in bone marrow^101^. The model partitions the cell cycle into quiescent and dividing phases, and a model parameter controls the transition between quiescence and proliferation. The study further analyzes the stability and persistence of the solution, offering conditions for global asymptotic stability and the existence of a unique positive steady state.

RD models are a specific subset of PDEs and share their computational challenges. Computing RD models involves the numerical solution of PDEs that describe the diffusion and reaction processes. Researchers have applied different techniques to obtain the exact and numerical solutions to these problems^109,110^.

### Boolean models

In the study of cell cycle dynamics, Boolean networks use nodes to represent genes or proteins and edges to indicate interactions, where each node is simplified to a binary on/off state. This approach efficiently identifies stable states or attractors corresponding to biologically significant phenotypes, providing qualitative insights and circumventing parameter estimation challenges common in ODEs-based models. Both Boolean and ODE models converge on the understanding that the cell cycle behaves as a limit cycle, a stable, repetitive sequence of events^111–113^. While the previously discussed modeling techniques can be implemented using standard programming languages, the implementation of the Boolean model is challenging. Therefore, various software tools featuring different mathematical and computational methods have been developed to simulate Boolean models, including MaBoss^114^, GINsim^115^, BoolNet^116^, SQUAD^117^, etc. Notably, MaBoss employs a continuous-time Markov process, and SQUAD utilizes network conversion to differential equations, which enables the generation of the temporal evolution of biological processes from a Boolean model. While Boolean models allow for the identification of stable cellular states, they face limitations in detailing the transient kinetics that lead to these endpoints. The capabilities of MaBoss and SQUAD to simulate temporal evolution offer practical solutions for filling the gap between qualitative and quantitative modeling.

### Agent-based models (ABMs)

ABMs offer a modular, mechanistic framework for simulating the evolution of behavior and phenotype switches of individual cells. These models can operate within structured environments, such as a two-dimensional or three-dimensional mesh, including lattice-based cellular automata models, or within more flexible, gridless spaces (off-lattice model)^118,119^. This setup enables detailed simulations of cell-cell interactions, diffusion of molecules, and the impact of therapeutic interventions. The flexibility of ABMs allows for the coding of computational rules that govern cellular phenotypic decision-making, facilitating simulations of how cells interact amongst themselves and with their microenvironment.

ABM’s capability to track the spatiotemporal distribution of tumor cells with different phenotypes is particularly valuable. These phenotypes can be discrete, representing distinct cellular states, such as cell cycle phases, cells of distinct metabolic activity, cells of distinct mitotic potential, hypoxic cells^120–124^, or can be continuous, detailing attributes like cell proliferation time or cell cycle division time^125^. ABM simulations, accounting for a tumor’s microscopic composition, illustrate how macroscopic patterns of cell organization are intrinsically tied to emergent cell behavior and microscopic interactions^126^. This approach highlights phenomena such as the way a mutation in a single cell can impact the growth dynamics of the whole tumor tissue^127^.

### Choosing the modeling paradigm

In this section, we compare deterministic and stochastic models, single-cell versus population models, and mechanistic versus abstract models to identify the most appropriate modeling framework for different research objectives.

Single-cell computational models utilize techniques such as ODEs, PDEs, and RD models to detail molecular mechanisms influencing cellular decisions, offering a granular view of biochemical reactions that operate closer to the actual dynamics of drug actions and cellular network complexities. On the other hand, population-level models use similar mathematical approaches but focus on capturing inter-cell variability in physiological states and cell cycle progression. By incorporating phenotype evolvability, these models can simulate tumor adaptation to selective pressures, such as immune surveillance, hypoxia, and therapy. This capability offers valuable information for developing strategies to combat drug resistance and manage tumor heterogeneity. The choice between population-level and single-cell models depends on the research objectives: single-cell models are optimal for detailed molecular insights critical for identifying therapeutic targets, while population models are better suited for exploring broad tumor dynamics and evolutionary pressures, aiding in the design of comprehensive treatment strategies.

In modeling the cell cycle, selecting between deterministic and stochastic approaches or integrating both depends on the specific aspect of cell biology under investigation and the desired scale of analysis. Deterministic models are widely used for studying average behaviors of cell populations and regulatory networks, offering predictions that align with bulk experimental data. However, they may not capture individual cell variability as they assume homogeneity within cell subclones. In contrast, stochastic models allow for the exploration of how individual events, like the emergence of resistant phenotypes, can lead to broader population trends. These models account for intrinsic random processes like gene expression and mutation variability that underlie the cell cycle control mechanisms^128^. Both approaches face challenges in model parameterization. ODEs and PDE-based optimization may encounter non-convexities that risk getting stuck in local minima, while stochastic ABMs and other stochastic simulations might struggle with scalability and the computational burden of simulating noisy systems. This complicates the search for globally optimal solutions and necessitates advanced statistical methods for robust analysis^129^.

Hybrid modeling achieves accuracy and computational efficiency by integrating deterministic and stochastic schemes within a single framework and benefits from the multiscale characteristic of biochemical systems with inherent variability in reaction rates and reactant populations. Techniques like the hybrid method by Haseltine and Rawlings^130^ and the approaches proposed by Salis et al.^131,132^ partition a system into groups of slow and fast reactions. Liu et al. proposed a new hybrid stochastic cell cycle model that outperforms purely stochastic models in terms of computational efficiency, efficiently simulating the stochastic nature of cellular processes^133^. Bouhaddou et al. developed a hybrid model that integrates stochastic processes, such as gene switching and mRNA synthesis/degradation, with deterministic biochemical reactions. This multi-omics tailored model offers insights into the contextual control of cell proliferation and death in response to drugs and mitogens^134^.

Mechanistic models, grounded in the physical, chemical, and biological principles underlying the system of interest, focus on how system components interact and respond to changes by incorporating causal relationships and processes governing system behavior^135^. These models are particularly chosen when the goal is to explore the fundamental mechanisms of a system, to predict outcomes under novel conditions, or to make predictions beyond the original data^136^. However, the complexity and the need for detailed system knowledge often make them challenging to construct and computationally intensive to analyze, which limits their use in scenarios with incomplete data or complex systems. In contrast, abstract models, including statistical, data-driven models, and ODE models with a high level of abstraction, emphasize capturing relationships and patterns within the data, often without addressing the underlying biological or physical mechanisms. These models can be highly effective in scenarios where detailed system knowledge is lacking, when the focus is on prediction rather than explanation, or when dealing with high-dimensional data from sources like genomics. The ability of abstract models to identify patterns within complex datasets makes them useful for hypothesis generation and the identification of potential areas for mechanistic study. Nonetheless, the lack of a direct link to underlying mechanisms may limit the interpretability of these models and their ability to provide mechanistic insights. Furthermore, abstract models may struggle to predict outcomes for conditions outside the original data, which poses challenges in extrapolating findings to novel scenarios^137^. Increasingly, hybrid models that combine elements of both mechanistic and abstract approaches are being developed to leverage the strengths of each, offering a powerful strategy for tackling the complexities inherent in biological systems.

### Bridging scales in biological systems through hybrid multiscale modeling

Cellular behaviors have significant impacts on tissue and organ-level outcomes, necessitating models that can navigate these complexities across different scales. Single-scale modeling approaches often fall short of capturing the multi-dimensional intricacies of biological systems, particularly due to their limitation in addressing only one aspect of system complexity at a time. Hybrid multiscale models, especially in fields like oncology^138^, bridge the microscale of the cellular dynamics and the macroscale of the population trends. These models are able to cover a wide range of biological aspects, from molecular interactions and cell evolution to tissue dynamics and patient-specific responses, enhancing model credibility through comparison between model output and experimental and clinical data. They integrate deterministic and stochastic approaches to accurately study molecular interactions’ dynamics and cellular behaviors’ randomness. Molecular-level insights are provided by molecular dynamics simulations, while cellular processes are elucidated through agent-based models and cellular automata, highlighting the intricacies of cell evolution and population dynamics. At a larger scale, continuum models utilizing PDEs address tissue dynamics, encompassing diffusion, cell proliferation, and mechanical forces. Such comprehensive coverage enables a deeper understanding of biological phenomena, including the extracellular matrix’s role in lumen morphogenesis^139^, cancer therapy through combinations of macrophage-based, hypoxia-targeted gene therapy and chemotherapy^140^, and the development of personalized cancer treatments that consider both inter-patient heterogeneity and intra-tumoral variability^141^. However, multiscale models sometimes face challenges in fully integrating cellular networks or functionalities, such as the cell cycle, metabolism, signaling cascades, and gene regulation networks. This is often due to the inherent complexity of these systems and the difficulties associated with scaling detailed interactions to higher organizational levels. Efforts to enhance these models include refining computational techniques and incorporating more comprehensive datasets.

Recent advancements in open-source multiscale models have significantly reduced technical hurdles, making software more accessible to researchers from a variety of disciplines. A standout example is the Theatre for in silico Systems Oncology (TISON), which has made remarkable progress by providing a “zero-code” environment. This platform enables the design of personalized cancer therapeutics through the integration of scale-specific information, spanning from molecular interactions to organ-level dynamics^142^. Alongside TISON, tools such as PhysiCell^143^, PhysiBoss^144,145^, Morpheus^146^ exemplify state-of-the-art multiscale modeling frameworks that are scalable and open-source. They are designed to be integrated with other modeling techniques for a more nuanced understanding of specific diseases. These platforms allow for the detailed modeling of the cell cycle, with the capability to show how alterations at this scale can propagate to tissue growth or mechanics on larger scales.

### Linking models to experimental and clinical data

Models are essential in bridging theoretical frameworks with empirical data as they explain observed trends and forecast future behaviors. They fulfill two principal roles: explanatory and predictive. Explanatory models are designed around specific experiments and are used to rationalize the experimental results and test hypotheses. Predictive models formulated based on the theoretical frameworks extrapolate from the given states to predict how cells might behave under new sets of conditions^147^.

**Fig. 4:**
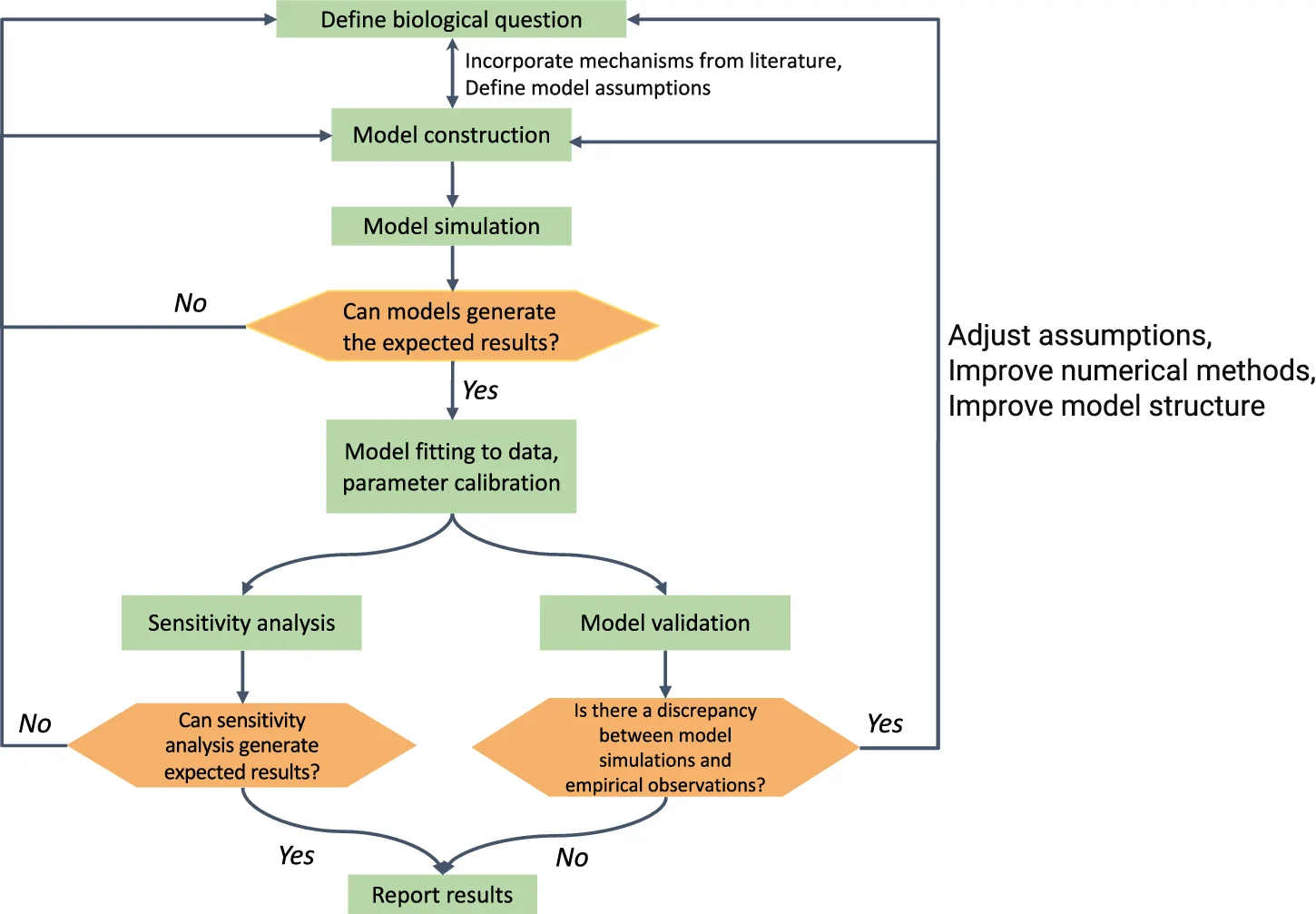
Flowchart for computational model development in systems biology. This flowchart shows the steps in computational model development, from defining a biological question to model construction, parameter fitting, and validation. Adjustments are based on simulation results and empirical data discrepancies.

The synergy between computational and empirical approaches fosters a robust iterative process^148,149^. In this paradigm, in silico models utilize distinct types of data to inform and guide experimental designs. ODE models primarily utilize time-series data that quantitatively capture dynamic changes, such as concentrations of reactants and products in kinetic studies or population changes. Probabilistic models leverage a variety of data types, including experimental measurements with inherent variability and historical datasets with trends and distributions, to analyze stochastic events such as gene expression noise or cellular response variability. Machine learning models depend on varied, large datasets such as genomic sequences and clinical biomarker data to predict complex biological behaviors like disease progression or treatment outcomes. PDE models analyze spatial and temporal data to model phenomena such as the dynamic spatial distribution of signaling molecules. Feedback from in vitro and in vivo experiments contributes to the refinement of in silico models and to the validation of the hypothesis generated by the in silico models. The iterative feedback loop between experimental studies and model refinement (Fig. 4) ensures that computational models evolve in alignment with empirical insights, enhancing their accuracy and applicability.

The transition from preclinical research to clinical application necessitates an expansive and multifaceted computational approach, integrating diverse data sources and methodologies to capture the complexity of individual tumors. Incorporating patient-specific biomedical images, permitted by advanced imaging analysis techniques like radiomics and digital pathology, into the construction phase of models for personalized cancer therapy plays a crucial role in enhancing the accuracy and relevance of these models. This process allows for a tailored approach where, in stochastic models, the initialization can leverage image-derived features to offer a patient-specific starting point over a randomized one^150^. Both deterministic and stochastic models utilize patient-specific images to fine-tune their parameters, with techniques being developed to calibrate ABM models to real-world data, ensuring that the models are closely aligned with the actual physiological conditions of the patient’s cancer^151–153^. Integrating genomic, transcriptomic, and proteomic data from tumors can help precisely identify the specific mutations and alterations driving cancer progression, pinpoint molecular targets for therapy, and accurately predict patient responses to specific treatments^154,155^. The use of longitudinal data from electronic health records significantly enhances the dynamic modeling of cancer progression, adapting models in real time based on individual treatment responses^156^. Pharmacokinetic/pharmacodynamic (PK/PD) modeling further optimizes dosing strategies by understanding the dynamics of drug action and metabolism, which is crucial in the administration of chemotherapeutic agents. With the advent of immunotherapies, modeling the immune system’s interaction with cancer cells becomes indispensable, offering predictions on individual responses by simulating intricate tumor-immune interactions. Together, these methodologies enhance the translational potential of computational models from preclinical to clinical stages.

### Enhancing computational model precision through parameter estimation, sensitivity analysis, and validation

Parameter estimation and sensitivity analysis are crucial for enhancing the robustness of computational models, particularly in biomedical research. By accurately estimating parameters, these models align more closely with real-world biological processes, such as tumor growth kinetics under various treatments, thereby increasing their applicability in clinical settings. Sensitivity analysis further aids this alignment by rigorously determining how variations in parameters affect model outcomes, underscoring the parameters’ functional role in model adaptation^157^. Additionally, these parameters clarify the quantitative behaviors of the underlying biological processes, revealing the complex dynamics at play. Such analysis is vital for both validating the models and for fine-tuning them to capture essential behavioral effects qualitatively and quantitatively. Validation is achieved by comparing model predictions against experimental data through methods like visual inspection and statistical tests, including likelihood and root mean square error (RMSE)^158^. This evaluation ensures that the models can reproduce clinical outcomes across various treatment strategies, highlighting the interpretive power of the parameters.

Parameter estimation is essential in computational modeling for identifying parameter values that optimally describe behavioral data. This process is typically approached through optimization techniques and sampling methods. Optimization involves iteratively adjusting model parameters to optimize an objective function, like least squares or Bayesian likelihood and posterior, through gradient-based methods or stochastic approaches. The choice between these methods depends on factors such as computational speed, model complexity, and the need for accuracy versus speed, as discussed in^159,160^. Sampling approaches like Markov Chain Monte Carlo (MCMC) navigate the parameter space differently by sampling point estimates from the posterior distribution using Bayesian inference principles. The statistical summary of these estimates quantifies the uncertainty and variability of parameter calibration and assesses confidence in model predictions. This method is particularly useful in complex models where traditional optimization might struggle. For a deeper dive into the varieties and applications of MCMC techniques, the review by Luengo et al. provides an insightful overview^161^.

Sensitivity analysis is a useful tool for testing the robustness of modeling under uncertainties, aiding in model refinement and the planning of future experimental designs. This analytical method quantitatively assesses how variations in input parameters influence the variability of model outputs. By doing so, it facilitates the determination of the model’s stability and identifies critical parameters that significantly impact the results. These insights are key to ensuring models accurately reflect real-world phenomena. Several techniques have been developed for sensitivity analysis, each suited to different types of models and objectives. Local sensitivity analysis evaluates changes in the model outputs with respect to variations in a single parameter input. In contrast, global sensitivity analysis assesses the effect of varying all parameters over the entire parameter space, which allows us to simultaneously evaluate the relative contributions of each parameter as well as the interactions between parameters to the model output variance. Established methods such as the Sobol method, multi-parametric sensitivity analysis, and the Morris method are commonly employed, each offering unique advantages in decomposing output variance and identifying influential parameters^162^.

## Applications of cell cycle models to model biological phenomena

### Modeling cell cycle reaction network centering on cyclin/CDK complexes

The modeling of regulatory networks in the cell cycle has been extensively studied in the literature. Some models focus on localized processes within the cell cycle, particularly the feedback loops crucial for regulatory functions^163^. These feedback loops are instrumental in stability analysis, a critical approach that analyzes how the cell cycle responds to genetic or molecular changes to maintain stability. Some models address specific cell cycle phases and transitions^64,164–166^, while others aim to comprehensively describe the regulatory system governing the entire cell cycle. The cell cycle regulatory models have been proposed for yeast and mammalian cells.

Due to its rich dynamical properties, the cyclin/CDK network has been extensively modeled using both ODEs and stochastic methods. Novak, Tyson, and their colleagues employed numerical integration techniques, such as temporal simulations, phase space analyses, and bifurcation diagrams, laying the groundwork for elucidating dynamic transitions in the cell cycle and how it progresses and responds to various signals^63,64,167^. Building on this theoretical foundation and focusing on the cell cycle’s temporal self-organization, Gérard et al. proposed a more integrative model featuring four cyclin/CDK modules corresponding to distinct cell phases^65^. The model takes into account endoreplication, a process in which DNA replicates multiple times without undergoing mitosis, and uses kinetic equations of protein levels to examine the G1 restriction point and the oscillatory balance between pRB and E2F. It also highlights how incorporating DNA replication checkpoints mediated by kinases ATR and Chk1 can slow the dynamics of the cell cycle while preserving its oscillatory nature and enhancing the separation between the S and M phases. Subsequent refinements further explored cell proliferation’s regulation by environmental factors^168^ and the feedback loops’ role in maintaining CDK oscillations’ robustness against molecular noise^66,67^. Yang et al. used a PDE model to examine protein translocation between cytoplasm and nucleus, which couples the mechanism of cell division to cell growth^169^. Weis et al. combined two existing models to better align model dynamics with quantitative expression data, which identifies the previously unmodeled dynamics as dominant factors controlling the dynamic expression profiles of cyclins A2 and B1^170^. Hernansaiz-Ballesteros et al.’s study based on the ODE model and bifurcation analysis revealed that the atypical interaction between Wee1 kinase and Cdc25 phosphatase with CDKs is crucial for maintaining G2 phase stability and cell cycle checkpoints. Their phylogenetic analysis suggests that this mechanism, present since the last common eukaryotic ancestor, may have been pivotal in eukaryotic evolution^171^.

Transitioning to stochastic methods, recent efforts in constructing stochastic models of the CDK control system in budding yeast have laid down a solid theoretical framework, emphasizing how feedback mechanisms within the cell cycle’s regulatory network help maintain molecular regulator fluctuations within manageable limits^172,173^. This is in line with findings from ODE models, underscoring the critical role of feedback in maintaining cellular stability. Moreover, Mura et al. introduced a stochastic Petri Nets (SPN) version of Novak and Tyson’s deterministic model^174^. This novel approach not only corroborated the deterministic simulation results but revealed unique budding yeast cell characteristics not observed in the deterministic version^175^. The stochastic simulations by Gerard et al. based on their skeleton model in ref. ^67^ suggest that stochastic switches between cell cycle arrest and proliferation may provide a source of heterogeneity in a cell population, as observed in cancer cells characterized by CDK1 overexpression^176^. The aforementioned CDK regulatory models can be incorporated as subcellular signaling components into larger-scale agent-based models. This integration, as demonstrated in studies by Zhang et al.^177^ and Wang et al.^178^, helps bridge the gap between cellular behavior at the microscopic level and macroscopic cellular phenomena.

Boolean network models have been proposed to systematically characterize behaviors aligned with the cell cycle regulatory graph^179^. Zhang et al.^180^ built a probabilistic Boolean network on the cell cycle protein interaction network in ref. ^181^. They characterized the noise as a temperature-like parameter that affects the temporal evolution pattern of the proposed Boolean network. They found that both the biological stationary state and the biological pathway are well preserved under a wide range of noise levels until reaching a critical threshold, beyond which the dynamics became noise-dominated. Davidich et al. built a ten-node network model of the fission yeast cell cycle regulation and found the model’s behavior converges on a limit cycle that reflects the oscillatory nature of the cell cycle. Additionally, they identified stable states (fixed points) within the network with G1 as the dominant one^111^. Deritei et al. advanced this with a logic-based model, which features “conditionally stable motifs”. These motifs maintain a consistent state, and their stability is contingent on the conditions set by the state of external nodes akin to biological cell cycle checkpoints^112^. In scenarios where these nodes remain locked, the model is transformed into an autonomous oscillator.

Singhania et al. took a hybrid approach, combining continuous differential equations with discrete Boolean networks to study the complex network governing cell cycle timings while circumventing the challenges of estimating kinetic constants^182^. Their model tracks cyclin abundances using piecewise linear differential equations, but the synthesis and degradation of cyclins are governed by discrete variables (0 or 1) representing transcription factors and ubiquitin-ligating enzyme complexes. These discrete variables change in a predetermined sequence with cyclins feedback on the discrete variables by determining how much time is spent in some of the Boolean states. The model’s robustness is affirmed by its alignment with human cell lines flow cytometry data.

### Models to study cell cycle variability

The growing cell populations, when analyzed at the single-cell level using techniques such as time-lapse imaging and flow cytometric methods, exhibit significant variability, spanning from genetic to phenotypic characteristics^183–185^. This variability is evident, for instance, in post-division sister cells where asymmetric division can lead to differing cell fates^186^. In studies measuring and analyzing the cell cycle in lineage trees of cells, intra-generational correlations in cell cycle duration have been observed. This correlation could be attributed to the inheritance of both cell size and cell cycle speed over several generations^187,188^. A counter-intuitive correlation pattern seen in many cell types is the “cousin-mother inequality” where the inter-division times of cousin cells are more correlated than those of mother-daughter pairs^188^. Hughes et al. established a mathematical framework for understanding how hidden factors inherited across cellular generations influence cell cycle timing. Utilizing Bayesian inference on single-cell datasets from bacterial, mammalian, and cancer cells, they analyzed the inheritance motifs impacting cell division. Their findings indicate that no single cell cycle model can be conclusively determined due to a broad posterior distribution of possible mechanisms. However, the correlation patterns in interdivision times reveal interpretable inheritance dynamics and hidden rhythmicity in cell cycle factors that are driven by circadian rhythms and may be disrupted in cancer^189^. Mathematical models navigate through the intricacies of cell size control, variable cell cycle completion times, and stem cell renewal dynamics to shed light on the fundamental question of “why” cell cycle variability exists and how it affects biological processes.

Significant progress has been made in recent years in quantifying the variability of cell cycle times, with the duration of each cell cycle phase often modeled as random variables. One classic model is the Smith-Martin model, which simplifies the cell cycle into two phases: exponentially distributed A phase and a fixed delay B phase^190^. An extension of such is a two-transition probability model to account for the responses of quiescent cells to stimulation by growth factors^191^. While these models stand out for their simplicity and mathematical tractability, the evolving complexity of cellular processes has led researchers to develop more detailed models that incorporate context-dependent physiological processes. This shift toward complexity is evident in the adoption of age-dependent models, where the focus expands to the behavioral history of each cell. Age-dependent cell cycle progression considers the impact of a cell’s age on its division behavior, acknowledging that as cells age, genetic and molecular shifts may influence their probability of division. Models like^192–195^ illustrate this diversity, exploring the relationship between individual cell division time variations and population-level fluctuations.

The multitype Bellman-Harris process models cell lifespans and genealogies from a single founding cell. This process has been effectively utilized in examining hippocampal neurogenesis^196^ and proliferation and differentiation of O-2A progenitor cells^197^. There is growing interest in incorporating cellular dependencies into the branching process framework, including aspects such as the inheritance of cell cycle lengths^198^ and inheritance of fate decisions^199,200^. The approach presented in ref. ^200^ assumes that all cells within the same clone undergo a predetermined number of divisions before they can differentiate or die. Variability in cell fate within the clone is introduced by randomly varying the number of division cycles across different clones. Hyrien et al. introduced a multi-type age-dependent branching process for analyzing cell kinetics during CFSE-labeling experiments, an experimental procedure to investigate the proliferative abilities of purified lymphocytes^201^. Their calculation demonstrates the model’s robustness with respect to cross-sectional (i.e., non-linearly filiated) dependencies and dependencies between fates of linearly filiated cells. However, they also note that it is unclear whether the cellular dependencies observed using timelapse microscopy experiments in clonal studies in vitro are applicable to in vivo simulations. This is because in vitro simulations cannot fully capture the physiological complexities of in vivo simulations, such as cell-cell contact. Additionally, they provided valuable quantitative analyses on CD8 T cell kinetics and insights into memory cell formation. Nordon et al. proposed a multi-type branching model, which is further modified to describe the inheritance of generation time and phenotype^202^. The cell generation time distributions follow the Smith and Martin model^190^ with a shift in the exponential distribution. The model with inheritance was fitted to mouse granulocyte-macrophage progenitors using live cell imaging data.

Comparative analyses by Zilman et al.^203^ and Miao et al.^204^ have highlighted the similarities and distinctions between probabilistic models like the branching process and deterministic approaches like age-structured models and ODE-based models. These studies elucidate how different modeling frameworks can influence the predicted cell counts and kinetics, reflecting the diverse outcomes possible when various theoretical perspectives are applied to generational cellular dynamics.

As introduced in Section ''Probabilistic models'', MSMs for a population of dividing cells are anchored in the Markov process and divide the cell cycle into sequential stages with transitions governed by exponentially distributed waiting times, leading to division times that follow an Erlang distribution. Based on work by Yate et al.^80^, Belluccini et al.^81^ incorporated death into the MSM model and derived the analytical solution for the expected number at each cell cycle stage across generations, assuming identical Erlang division times, consistent death rate, and uniform number of stages N across generations. This allowed for precise model calibration with CFSE labeling data. The refined MSM model aligns conceptually with the cyton model that describes proliferating lymphocytes governed by competing timers for cell division and death^205^. This alignment is maintained when the MSM model is simplified to a single stage, suggesting the broader applicability of these modeling approaches across different cellular contexts. The MSM is able to address experimental inconsistencies by modeling both the exponential growth of the overall population and the oscillations within subpopulations identified as inherent synchronization through fluorescent ubiquitination-based cell cycle indicator (FUCCI) imaging^83^. It is important to note that this inherent synchronization could impact the reproducibility of experiments designed to explore cell cycle-dependent mechanisms, such as alterations in cell migration and responses to drugs. Moreover, Perez-Carrasco et al. introduced a theoretical framework using MSM to examine how cell cycle duration and DNA replication timing variations affect mRNA fluctuations for genes expressed constitutively or in bursts. They highlighted that neglecting cell cycle effects can underestimate mean mRNA levels, with the degree of error tied to the ratio of cell cycle length to mRNA lifespan. Furthermore, they noted that the greatest discrepancies in mRNA variance occur at intermediate values of this ratio, consistent with global data from multiple organisms^206^.

### Modeling the balanced growth

Balanced growth characterizes a state in cell populations in vitro where, after starting synchronously, they achieve a steady state with constant phase distributions over time, assuming a stable, nutrient-rich environment^207^. In this case, the number of cells in each cell cycle phase expands at a rate that is proportional to their respective proportions within the total population, reaching steady-state proportions that are independent of initial conditions. Following the transient period of population adjustment, cell age distributions in each cell cycle phase are time-invariant during the balanced growth phase, leading to exponential population growth with a consistent rate, despite changes in individual cell sizes^208^. This condition is also known as asynchronous or exponential growth. Arino et al. demonstrated that the phenomenon of asymptotic exponential growth exists in a wide class of models^209^. Such a state of equilibrium in macroscopically homogeneous cell systems remains critical for understanding cellular dynamics and underlying nonlinear systems^210,211^.

The growth rate of the population and unchanging percentages while the population is in balanced growth can be solved using the dynamic system models. If the transition rates in an ODE system governing the dynamics of cell cycle progression are time-invariant, the constant densities of the phases will be the eigenvector corresponding to the coefficient matrix’s dominant eigenvalue^87,102^. For the systems where the transition rates are time-dependent, due to, for instance, the effect of circadian rhythms on cell cycle progression in physiologically structured models^212^, determining the population growth rate involves finding the eigenvalue of the matrix derived from the discretization of the original system.

The challenge in age-structured models with age-dependent transition rates lies in the difficulty of measuring transition age distributions. Although synchronizing cells and continuous observation are tentative solutions, they both have unique limitations. Sherer et al. ’s experimental and analytical approach overcome this by using BrdU labeling to distinguish between subpopulations during unbalanced growth^87^. This facilitates the estimation of transition rates from the transient period in which the cell cycle phases in subpopulations are evolving to eventual constant density. They explored various transition types to examine their quality of fitting the experimental data, finding cubic spline nodes and lognormal distributions as effective fitting methods, the former offering precision with fewer parameters.

### Cell size control

Cell size control plays a crucial role in cell fate determination and cell cycle progression. This control involves feedback mechanisms that link phase duration with cell volume, which varies among different cell types such as fission yeast, mammals, and budding yeast^213^. Both budding yeast (Saccharomyces cerevisiae) and fission yeast (Schizosaccharomyces pombe) serve as important model organisms for understanding the regulation of the eukaryotic cell cycle^214^. Their conservation of cell cycle machinery and straightforward culture conditions make them ideal for study. In budding yeast, asymmetrical division results in differently sized mother and daughter cells^215^. The mechanisms underlying the contribution of size control on the variability of the budding yeast cell cycle and fission yeast cell cycle have been investigated in experimental studies^216,217^. These investigations have inspired stochastic models that account for size control mechanisms in both mother and daughter cells in the budding yeast cell cycle^172,216,218,219^. Orlando et al. presented a fully parametric mathematical model studying the dynamic shifts in population distributions over time in budding yeast^220^. This model accounts for synchrony loss resulting from asymmetric cell division through a branching process structure. Barber et al. further explored these themes, focusing on the impact of single-cell variability on population growth rates^221^. Model simulations suggest that cells that divide asymmetrically and regulate their size effectively can inherit generation times through epigenetic mechanisms. These cells can also compensate for any deficits in growth rate through their size control during asymmetric division. Conversely, fission yeast undergoes symmetric divisions, with extensive mathematical modeling studies on the interplay between cellular growth and division with critical insights that may be generalizable to more complex biological systems^222–224^. Assuming cell division to be a random process with size-dependent probability, size-dependent probabilistic models of progress through the cell cycle have also developed^225,226^, among which the “sloppy” size control in ref. ^226^ is able to fit the division size histogram and the generation time histogram.

### Stem cell renewal dynamics

Stem cells maintain or expand their populations through their capacity for self-renewal, facilitated by both symmetric and asymmetric divisions^227^. Symmetric divisions produce either two stem cells or two differentiated cells. Stochastic models^228^ and mean field continuum model^229^ are developed to describe symmetric divisions of stem cells. Asymmetric division, where a stem cell divides into one stem cell and one differentiated cell, plays a crucial role in balancing self-renewal and differentiation, key for tissue development, repair, and maintaining homeostasis^230^. This process has been studied by various types of mathematical models^231–233^. Both symmetric and asymmetric divisions of stem cells are considered in a discrete state multiscale model, which includes a cytokinetic model for tumor response to chemotherapy^234,235^. They employed innovative techniques for initializing tumor compositions to avoid unrealistic growth patterns due to arbitrary model initialization. Furthermore, in vitro and in vivo studies have shown that similar regulatory pathways may control both cell cycle progression and differentiation across various biological systems^236–238^. Notably, Stopka et al. employed a stochastic model to assess how interactions between cell cycle progression and differentiation influence homeostasis at the single-cell level. This model assigns an intrinsic probability to stem cells for progressing through the cell cycle or for differentiating. Their analysis of publicly available single-cell RNA-Seq gene expression data from the adult mouse brain showed a substantial positive correlation between cell cycle activity and stem cell differentiation. These findings emphasize the integral role of this coupling in maintaining brain tissue homeostasis, highlighting its significant implications for advancing our understanding of neural development and regeneration^239^.

### Interplay between DNA replication and cell cycle progression

DNA replication is a complex process that involves the origin licensing, initiation (origin firing), elongation, and termination, alongside the coordination of multiple protein complexes and regulatory mechanisms to ensure accurate and complete DNA replication. In addressing the coordination between DNA replication and cell cycle progression, both stochastic models and deterministic mechanistic models are often used. The application of stochastic models in studying DNA replication is justifiable by the process’s inherent stochasticity with uncertainties in both the location and time of firing of replication origins across different cells and conditions. The genome-wide mapping study by Wang et al. showed that stochastic regulation of replication kinetics is a fundamental feature of eukaryotic replication, conserved from yeast to humans^240^. By incorporating a random selection of licensed origins and parameters such as the rate of origin licensing and the probability of origin firing, stochastic models are able to offer insights into variation in replication timing and efficiency across different cells, as seen in ref. ^241^. Some models consider the DNA replication initiation sites along the fission yeast genome^242–244^, and in human genome^245^, underscoring that the stochasticity in the activation of replication origins contributes to the variability in replication rates and overall duration of the S phase. Deterministic mechanistic models, on the other hand, focus on the biochemical and physical interactions that underpin the origin licensing and firing processes. These models are grounded in known molecular mechanisms and aim to elucidate how specific sequences of events lead to the initiation of DNA replication. For instance, Williams et al. constructed an ODE model that integrates aspects of cell cycle control, origin licensing, mechanisms to prevent rereplication, and kinetochore attachment^246^. The model is shown to be robust to parameter changes, and cycling is maintained despite parameter changes.

### DNA damage repair and cell cycle progression

Recent studies have advanced our understanding of the DNA damage response and repair mechanisms through innovative modeling approaches. Mohseni-Salehi et al. built a three-dimensional stochastic process using a continuous time Markov Chain to monitor the cell cycle phases and DNA double-strand breaks (DSBs) repair post-irradiation, highlighting the competition between nonhomologous end joining and homologous recombination repair mechanisms^247^. This model effectively simulates repair dynamics across the cell cycle and in response to various radiations. Tashima et al. constructed a kinetic model in which the DNA damage signaling pathway interferes with G2/M cell cycle progression, demonstrating how varying intensities of DNA damage can lead to cell cycle arrest^248^. Iwamoto et al. explored the DNA damage signaling pathway’s impact on cell fate through a model that includes p53 oscillation, emphasizing its role in cell fate selection^249^. Hodgkinson et al. utilized structural modeling to explore cell cycle dynamics at the level of the individual cell and population^250^. The cell level model uses a PDE framework to analyze cyclin levels individually and throughout the cell cycle. A spatio-temporal-structural population PDE framework helps understand how p53 protects the cell from the propagation of DNA damage. The model indicates that reduced DNA repair rates can hinder progression to later cell cycle stages due to a decrease in p53 levels in cells with extensive DNA damage. Hu et al. studied the cellular response to different linear energy transfer (LET) radiations by augmenting their previous model with modules for DNA repair and p53 regulation in cell cycle arrest and apoptosis^251^. They differentiated between fast and slow DNA repair pathways by considering the chromatin environment and DSB complexity. The simulation results highlight that DSB repair kinetics significantly influence cell fate, with complex DSBs induced by High-LET radiation resulting in prolonged cell cycle arrest, increased apoptosis, and elevated TGF*β* secretion. Mombach’s logical model provides an overview of how different levels of DNA damage lead to a specific cell fate. The possible outcomes are proliferation, transient DNA repair arrest, apoptosis, and senescence^252^.

### Models to explore the crosstalk between cell cycle network and signaling pathways in carcinogenesis

Leveraging mathematical models and in silico experiments, researchers are able to decode the intricate dynamics of signaling pathways and cell cycle dysregulation in carcinogenesis. These approaches enable the detailed examination of abnormalities in pathway components and their effects on cell fate decisions, shedding light on the processes that drive the malignant transformation of normal cells.

Recognition of the role of essential components in the signaling pathways is vital for understanding mechanisms. This includes understanding their hierarchy, modularity motif, and regulatory feedback loops within these pathways. By conceptualizing these relationships as circuit-like graphs, researchers can employ graph-based representations and logical models to simplify and analyze the complex dynamics of these networks over time, facilitating predictions about cellular behavior and responses^253,254^. For instance, Grieco et al. developed a comprehensive Boolean network model that studies the positive or negative influence of the MAPK signaling network on cancer cell proliferation, highlighting its role in cell cycle activation^255^. This model provides insights into the aggressive phenotype of bladder cancers through in silico experiments, revealing the importance of MAPK interactions and feedback mechanisms in determining cell fate. Moreover, focusing on the effects of hyperactive PI3K on cell cycle disruption, Sizek et al. introduced a Boolean network model of growth factor signaling that can reproduce PI3K oscillations and link them to cell cycle progression and apoptosis^256^. The resulting modular model predicts the molecular drivers responsible for cytokinesis failures by linking hyperactive PI3K to misregulation of Polo-like kinase 1 (PLK1) expression late in G2, while offering testable hypotheses on molecular drivers of PI3K oscillations and their impact on cell division.

The advancements in computational power and a deeper understanding of oncogenic pathways have significantly expanded the scale and sophistication of biological models, notably illustrated by the advancements in whole-cell models (WCMs)^257,258^. WCMs aim to predict cellular phenotypes from genotypes and the environmental factors by mapping out the function of each gene, gene product, and metabolite^259^, thereby offering a comprehensive view of cellular operations, including the cell cycle. A key aspect of WCMs is their focus on identifying hubs, i.e., elements with high connectivity in the cellular environment that serve as integration points for cellular networks^260^. Despite substantial advancements in mapping the cell cycle, challenges persist in fully elucidating the complexity. For instance, while major checkpoints and regulators have been thoroughly characterized, the nuances of their interactions, feedback mechanisms, and adaptive responses to various stimuli may remain ambiguous. Furthermore, the broad scope of WCMs presents significant hurdles in validating their accuracy, owing to the vast array of processes and interactions they encapsulate. Addressing these challenges necessitates further advancements in computational methodologies, enhanced integration of experimental data, and innovative approaches to corroborate model predictions with empirical evidence, paving the way for deeper insights into cellular behavior and cancer progression.

### Impact of hypoxia, nutrient deficiency, angiogenesis, mechanical forces in TME on tumor growth and cell cycle dynamics

An oxygen and nutrient-deficient environment is associated with strong selective pressure on tumor cells, favoring more aggressive clones with proliferative advantages^261^. Several mathematical models have been developed to understand these effects. Celora et al. proposed a mechanistic DNA-structured model focusing on cyclic hypoxia’s impact on cell cycle dysregulation^102^. This model successfully simulates increases in S phase duration, S phase cell accumulation, and G2 checkpoint arrest under cyclic hypoxia protocols, with predictions aligning with experimental observations. The model’s predictions for untested cyclic hypoxia protocols indicate a diverse range of cell responses depending on oxygen dynamics, highlighting the model’s potential to guide treatment optimization. In contrast to Celora et al.’s use of periodic functions to simulate oxygen levels, Powathil et al. employed RD equations to model the dynamic oxygen landscape^262^. Their model incorporates oxygen concentration as an inhibitory term that can arrest the cell cycle when levels fall below a certain threshold. Their simulations through a lattice-based, cellular automata approach reveal that proliferating cells predominantly occupy areas with ample nutrients and oxygen, whereas dormant cells are found in nutrient-poor regions. Shamsi et al. extended this understanding by exploring how the glucose metabolism of cancer cells influences tumorigenesis through a cellular automaton model^263^. Their model identified a specific range of glucose uptake rates at which glycolytic tumors appear most aggressive, exerting significant acidic stress on TME and efficiently competing for glucose supplies.

Before angiogenesis, tumor growth relies on the diffusion of nutrients through ECM. The nutrient concentration may affect the genetic signature of the tumor cells and the overall tumor growth. This avascular growth phase underscores the need for multiscale modeling to investigate the feedback loops across subcellular, cellular, and extracellular scales, reflecting the complexity of early tumor development. The multiscale model built by Jiang et al. integrated cellular dynamics, protein expression regulation, and TME nutrient dynamics^264^. Their approach, combining a lattice Monte Carlo model for cellular behaviors, a Boolean network for the expression regulation of proteins controlling the cell cycle, and RD equations for extracellular nutrient and growth promoter dynamics, predicts essential TME conditions supporting tumor cell survival and proliferation. Anderson et al.^265^ presented three single cell-based models to explore tumor initiation, growth, and invasion in the initial stages of tumor development at various biological scales. This framework comprises a Hybrid Discrete Continuum (HDC) model for cell-cell and cell-stroma interactions, an Immersed Boundary Cell (IBCell) model for cellular scale dynamics, and an Evolutionary Hybrid Cellular Automata Model (EHCA) on the subcellular scale that uses an artificial feed-forward neural network to simulate individual cell decision-making processes based on genetic and environmental cues. They also suggested possible ways to link the three models into a cohesive multiscale model to achieve a comprehensive understanding of early tumor dynamics.

As the tumor grows and adapts, it eventually reaches a critical size where it requires functional vasculature for nutrient delivery and further growth. Given the scope of this review, we focus on several multiscale models that can help elucidate how microvascular network growth promotes phenotypic heterogeneity. The study done by Zangooei et al.^150^ introduced a multiscale method combining PDEs, deep reinforcement learning (DRL), and ABMs to predict cancer and microvascular network growth patterns. DRL dynamically guides the decision-making process of each agent’s phenotype at every time step, allowing agents to adapt within an environment based on learned knowledge. The simulations show that as the difference between the expansion of the cancer cell population and the microvascular network increases, cells are more likely to undergo proliferation and migration than other phenotypes. Owen et al.^266^ adopted a multiscale model for vascular tumor growth, where an arbitrary vascular network adapts structurally to a variety of stimuli. RD equations model the spatial-temporal evolution of stimuli in the diffusible layer. This layer is connected with cellular and subcellular layers such that the dynamics of the intracellular phenomena are regulated by (and regulate) extracellular factors. Simulations indicate that the balance between vessel pruning and angiogenesis determines network remodeling and tumor growth, with vascular density and oxygenation levels regulating the transition from metastable states to rapid tumor expansion.

Mechanical forces and biophysical properties of TME are gaining attention due to their direct and indirect influence on tumor growth. These forces include cell-cell adhesion, ECM interactions, and basement membrane engagement, modeled in agent-based frameworks using potential functions. This modeling approach allows for the detailed study of cadherin-mediated adhesion, integrin-ECM bindings, and the mechanical constraints imposed by basement membrane contacts^267,268^. For example, Macklin et al. developed a lattice-free ABM for ductal carcinoma in situ (DCIS), incorporating biomechanical forces to simulate cell motion and tumor growth^269^. Calibrated with patient-specific histopathology, the model predicts that necrotic cell lysis acts as a biomechanical stress relief and is responsible for the linear DCIS growth observed in mammography. The continuum models, on the other hand, treat the tumor as a cohesive mass, applying continuum mechanics to examine the effects of tumor volume expansion and mechanical stress on tumor development. Elazab et al. proposed a modeling framework for cerebral tumor growth that couples diffusive and biomechanical models with treatment effects^151^. This model, leveraging modified RD mechanisms and continuum mechanics alongside an optimized use of medical image datasets, allows for the prediction of tumor growth and response to treatment.

### Models to study tumor-immune dynamics within the TME and immunotherapy outcomes

Modeling tumor-immune interactions and immunotherapy responses requires a comprehensive understanding of the dynamic interchange between tumor cells and the immune system within the TME. These interactions are often described through differential equations that detail cellular behaviors like proliferation, death, and differentiation in response to various internal and external stimuli^270^. ABMs are particularly useful in simulating heterogeneity and spatial dynamics of the TME^271^. These models incorporate key cell cycle parameters such as cell division and apoptosis rates, influenced by interactions with immune cells and TME factors like cytokines and growth factors. ABMs also account for the genetic and phenotypic heterogeneity of tumor cells. This includes various mutations that alter cell cycle regulation and immune evasion capabilities. A significant advancement in this field is the development of the spatial quantitative systems pharmacology (spQSP) model, a 3D hybrid platform that integrates quantitative systems pharmacology (QSP) models with ABMs. This innovative tool is versatile for analyzing responses to immunotherapy^272,273^. For example, in a study by Nikfar et al., the effects of intratumoral heterogeneity on the outcomes of anti-PD-1 therapy were investigated using this approach. By using spatial metrics from digital pathology and a non-spatial metric of cancer to immune cell ratios, they identified “cold”, “compartmentalized”, and “mixed” TME types. Their findings show that compartmentalized TMEs tend to have better treatment outcomes, consistent with clinical observations^274^. Bergman et al. developed a 3D multiscale ABM to examine the impact of fibroblast growth factor receptor 3 (FGFR3) mutations on immune responses and the efficacy of combined anti-FGFR3 small-molecule inhibitor (SMI) and anti-PD-1 antibody treatments^275^. Model simulations, which are based on parameters derived from existing literature, found that antigenicity and FGFR3 mutations influence disease progression and treatment efficacy, with treatment success dependent on the FGFR3 pathway’s activity. Similarly, Kather et al. applied ABMs to study the dynamic response to stroma-targeting therapies^276^.

## Enhancing cancer treatment strategies through computational modeling of the cell cycle

The chemotherapeutic drugs, often used as first-line treatment for various types of solid tumors, can be divided into several types, each of which targets a specific process within the cell cycle, such as topoisomerase action, synthesis of cellular enzymes in DNA synthesis, DNA damage response, nucleic acid metabolism or microtubule assembly^277,278^. Chemotherapy agents that target particular phases of the cell cycle are termed cell cycle-specific agents (examples include antimetabolites and taxanes). On the other hand, compounds that function at any stage of the cell cycle, including the resting phase, are labeled as cell cycle-non-specific agents (like alkylating medications and platinum derivatives, including cisplatin). A different treatment modality, ionizing radiation (IR), primarily damages DNA either directly through radiation or through the generation of free radicals^279^. The radiosensitivity of cells is also phase dependent, with cells in the G2/M particularly sensitive to ionizing radiation^280^. Both cytotoxic chemotherapy and radiotherapy are DNA damage-inducing therapies, albeit with distinct mechanisms of action^281^. Combinations of chemotherapeutics with varying DNA damaging mechanisms show synergistic and additive antitumor effects.

Alternatively, the combination of inhibitors of cell cycle proteins and chemotherapeutic drugs can enhance the sensitivity of tumor cells to the cytotoxic effects of chemotherapy^278,282^. The synergistic effects of such combinations have been proven in preclinical trials (e.g., ATR inhibitors and carboplatin^283^, Chk1 inhibitors and gemcitabine^284^). The relationship between cell cycle phase sensitivity and synergistic or antagonistic drug combinations has been widely studied^280,285,286^, promoting the development of effective drug combinations.

The development of effective cancer treatment is complicated by tumor heterogeneity, lack of predictive biomarkers, the occurrence of drug resistance, preclinical model limitations, etc. Computational models have emerged as powerful tools in response to the calls for automated process development^287^. In fact, the number of publications on the mathematical modeling of cancer is growing at an exponential rate^288^. The model-informed drug development (MIDD) approach spans the life cycle of the development of new drugs and informs clinical trials^289^. These models facilitate a reduction in design space, enable integration across multiple physiological scales, address drug resistance challenges, and aid in identifying globally optimal treatment protocols more efficiently and cost-effectively. Furthermore, the mathematical model can optimize mammalian cell culture bioprocessing essential for the production of biopharmaceuticals^290^, e.g., batch culture GS-NS0 cells used in the commercial production of monoclonal antibodies (mAbs)^291^ and Chinese hamster ovary cells (CHO) cultivated in fed-batch culture producing a therapeutic antibody^292^. By leveraging the quantitative power of mathematical modeling and advancing parallel computing capabilities, we can accelerate scientific discovery, optimize design choices, and make informed decisions. This approach allows for a detailed exploration of various dosing scenarios with varying schedules and dosages and it aids in determining the optimized protocol for antitumor therapies.

Throughout the rest of Section ''Enhancing cancer treatment strategies through computational modeling of the cell cycle'', we explore various computational approaches that have been employed to study the effect of cell cycle-dependent anticancer treatment on proliferation dynamics of the cancer cell population and to investigate emergent drug resistance. We also discuss the implications of these findings for the discovery and optimization of therapeutic interventions.

### Integrating cell cycle regulation in modeling the treatment efficacy

Quantifying the efficacy of therapies targeting the cell cycle and tumor heterogeneity involves delving into the finer partitioning of proliferating cells into subclasses during different phases (G1, S, G2/M) and considering treatment-induced cell types like apoptotic and resistant cells. Changes in phenotype triggered by treatment can be represented by modifying transition rates between different states in the deterministic model or adjusting transition probabilities in stochastic models. These shifts in modeling dynamics, driven by the pharmacodynamics of drugs, are critical for capturing the cellular responses to treatment. These models can easily accommodate drug combinations, reflecting different extents of agent interactions (e.g., additive, antagonistic, or synergistic) as anticancer drugs with different mechanisms are often given in different temporal sequences or concurrently. Through these models, insights into dose-effect relationships can be derived, offering guidance for optimizing treatment regimens. Sophisticated modeling techniques, such as those based on partial differential equations or agent-based approaches, embed spatial information to address the spatially varying drug concentrations. This acknowledges the fact that drug distribution is rarely uniform across tumor dimensions.

While the full biochemical network of the cell cycle is not always modeled in treatment efficacy models, incorporating phenotype transitions associated with therapeutic actions, such as DNA damage repair, replication stress, cell cycle checkpoint activation, and apoptosis, can enhance the model’s predictive power on how the anticancer drugs act on the tumor growth potential and how they induce cell death. This incorporation not only enriches the complexity and depth of the model but also bridges the gap between the macroscopic clinical outcomes and the microscopic cellular processes. Such phenotypic transitions are inherently governed by underlying biochemical networks where the drug operates, ensuring that the model retains core prior logical constructs^293^. It is noteworthy that some models opt to bypass the detailed kinetics of DNA damage measured by *γ*H2AX foci, a marker of chromosomal DNA damage. Instead, these models might generally represent the elongation of specific cell cycle phases caused by treatment-induced damage to a particular phase of the cell cycle.

### Application of computational models to quantify efficacy of treatment targeting cell cycle

Here, we explore the adoption of mathematical models to quantify the efficacy of treatments, highlighting their impact on predicting treatment responses and optimizing therapeutic strategies. Our discussion covers various models, including ODE models, PSPMs, mechanistic-based PK/PD models, ABMs, and deep learning models.

#### ODE models

ODE models have been employed to quantify the efficacy of therapies that aim to disrupt the cell cycle of cancer cells. These models track temporal changes in concentrations of cell cycle components under the influence of therapeutic agents. As it is not a physiologically structured model, these models segment a heterogeneous cell population into distinct compartments, each representing a uniform phenotype, to accurately depict phenotypic variability. For instance, Simms et al. dissected the cell cycle into seven model phases, including the storage phase (a mandatory amount of time) and non-storage phases (to study variability in cell cycle progression) for individual typical cell cycle phases^69^. They detailed the G1 phase’s responsiveness to environmental factors and included cell death mechanisms to simulate tamoxifen effects on breast cancer. Abroudi et al. took a multi-level systems approach that incorporated all known aspects of the cell cycle, revealing intricate details of cell cycle dynamics under both normal and DNA damage conditions^70^. The global sensitivity analysis identifies the most effective parameters, modules, and sub-systems on system response. Along the same lines, Alkan et al. linked chemotherapy-induced DNA damage responses to cell fate through a multiscale model, offering mechanistic insights into the effects of combined chemotherapy and DNA damage response inhibitors^294^. Bae et al. presented a model for FUCCI analysis^295^. Built on a two-ODE framework for CDK1 and anaphase-promoting complex (APC) activities with feedback mechanisms, this model incorporates stochastic elements to account for variability in cell cycle duration and examines the effects of treatments such as MLN8237 and nocodazole on cell cycle progression.

#### Physiologically structured population models (PSPMs)

PSPMs have improved our understanding of the drug effects on cell cycle dynamics by incorporating physiological variability. We focus on age-structured and DNA-structured PSPMs as they are considered effective models for simulating the evolution of the cell cycle and its response to drugs. Additionally, we will discuss the use of PSPMs in studying generational variations in drug response.

In the age-structured models, the introduction of cell cycle targeting treatments often alters the transition rates between cell cycle phases in an age-dependent manner^296^. For example, Bassie et al. developed an adaptable age-structured model for analyzing the impacts of various cancer therapies on a multi-compartment cell population model^297^. This model considers an unperturbed cell population in an asynchronous exponential growth state until the introduction of the therapy at a specific point. They suggested changes to the transition rate functions and the incorporation of additional phases of the cell cycle to reflect the impact of drug treatments. Considering that the errors present in the estimates of cell phase distributions may impact the simulation of treatment efficacy and planning, Chaffey et al. further examined how different transition rules at the G1-S checkpoint could affect cell phase distributions^298^.

Age can also be conceptualized as a dimensionless maturation index, ranging from 0 (newly formed cells) to 1 (ready for phase transition) rather than reflecting the actual time spent in a certain phase. This conceptualization necessitates a biologically realistic determination of the rate at which cells mature and advance through the cycle phases. Pisu et al. tackled this by employing a population balance model, which incorporates cell volume and age and dictates phase transitions based on the kinetics of underlying biochemical reactions^299^. In particular, the duration of each phase, defined as the inverse of the rate of change along the coordinate of age, is estimated by considering the progressions of specific biochemical reactions until checkpoints for transitions are reached. The model simulations demonstrate the ability of the proposed model to reproduce the slowing down of the cell cycle progression measured in in vitro after cytostatic drugs.

The cell cycle PSPMs can be structured based on the DNA content, a crucial aspect when understanding how cells progress and divide^102,300–302^. In this type of model, cells in the G1 and G2/M phases have constant DNA content (x), with x = 1 (original DNA content for G1) and x = 2 (doubled DNA content after S), respectively, while x increases at a constant rate for cells in the S phase. Building upon the foundational work by Basse et al.^300^, Lonati et al. developed a DNA-structured model to analyze the distribution of cells post-radiation exposure across different cell cycle phases for varying doses and times after exposure^303^. The modification of transition rate parameters allows for the reproduction of cell cycle perturbations induced by radiation.

The population balance model can characterize the varying effect of treatment across generations. Falcetta et al. developed a discretized population balance model of the cancer cell cycling process following X-ray exposure, providing separate and quantitative measures of the dose-dependence of checkpoint activities in subsequent generations^304^. The effects of treatments were modeled with modules describing the functional activity of the main pathways causing arrest, repair, and cell death in each phase. In a subsequent study, they calibrated this model using tumor growth data observed under various cell cycle-specific drugs and their combinations, allowing an in-depth comparison of the different treatment options^305^.

#### Mechanistic-based PK/PD models

The PK model details the drug’s movement through the body, focusing on its absorption, distribution, metabolism, and excretion. Conversely, the PD model examines the pharmacological alterations induced by the drug, illustrated by observable changes in physiological metrics like tumor volume or white blood cell counts post-treatment^306^. The PD model can be thought of as the result of structural modifications to the baseline model. Hence, as drug effects wear off, the tumor population will regain the proliferative momentum and return to the baseline level. As opposed to the population PK/PD model, whose aim is to predict treatment outcomes at the population level by taking into account patient characteristics and demographics, mechanistic-based PK/PD leverages knowledge about the drug-target interactions and physiological impacts, particularly on tumor growth^307^. By incorporating molecular details for intracellular drug effects and phenomenological representations for the cell cycle and tumor shrinkage, mechanistic-based PK/PD models provide a quantitative framework for understanding the drug’s actions and assessing the dose exposure-response relationship. Mechanistic-based PK/PD models provide a generalizable modeling platform for assessing antiproliferative drugs’ efficacy and exploring combinations of cytostatic and cytotoxic agents in cancer therapy. Research on CDK4/6 inhibitors, such as LY2835219, highlights their role in blocking the cell cycle’s progression by inhibiting CDK/cyclin complex activity and RB protein phosphorylation^308^. With a focus on the pharmacological action of one such inhibitor, LY2835219, Tate et al. proposed a semi-mechanistic PK/PD model integrating PK model, CDK 4/6 inhibition, cell cycle arrest, and tumor growth inhibition (TGI) submodels^309^. The novelty of this model is that they incorporate the biomarkers p-RB, TopoII*α*, and pHH3 to describe the cell cycle arrest and its effect on tumor growth kinetics. Similarly, Ma et al. connected the 5-Fluorouracil mechanism of action with tumor shrinkage through DNA damage markers, revealing the influences of DNA damage induction on tumor shrinkage^310^. Exploring drug combinations, Miao et al. analyzed the gemcitabine’s and trabectedin’s synergistic or antagonistic effects on cell cycle^311^, while Zhu et al.’s model evaluated the combined impact of gemcitabine and birinapant on cell cycle arrest and apoptosis^312^. Jackson et al. used a cell cycle model to explore cyclotherapy as a strategy for targeting defective cancer cell checkpoints, assessing how the G1-S and spindle assembly checkpoints impact drug selectivity^313^.

#### Agent-based models (ABMs)

Existing ABMs of tumor growth differ in their emphasis on subcellular processes of cell division, cell-cell interactions, cell movement, nutrient delivery, biomechanics, and whether or not they include the spatial distribution of drug concentrations. The coupling across scales in these models provides a comprehensive view of tumor behaviors under selection forces of treatment. For example, in a study by Powathil et al., subcellular processes were described using an oxygen-regulated cell cycle model^262^. This research reveals that when a fraction of cells are eliminated by the administered cell cycle-specific drugs, the surviving cells undergo a spatial redistribution. This redistribution consequently alters the dynamics of oxygen and drug concentrations, potentially impacting subsequent drug delivery. The combination of PK/PD and ABM provides more insights into the details of drug dynamics and the resulting impact on tumor growth^314^. In a study by Xie et al., a novel three-dimensional hybrid computational model was developed to analyze invasive solid tumor growth under chemotherapy in varied microenvironments^315^. The model reveals that constant drug dosing was generally superior in curbing primary tumor growth than periodic dosing, though this did not always inhibit invasive cell migration. Notably, the model’s core component, coupling a cell automaton model with a diffusion-reaction equation, is versatile and believed to be apt for simulations in anticancer drug screenings, particularly with tumor spheroids in vitro.

In the ABMs, the drug’s mode of action on cellular agents can be simulated in various ways. This approach includes integrating targeted drugs into the ODE model at the single-cell level, setting rules for the transition to treatment-induced death^262^, and incorporating treatment-specific parameters deciding state transitions. These parameters can dictate state transitions such as the propensity of quitting the cell cycle^316,317^, determine the death probabilities or mortality rate^120,125,318^, and adjust the progression speed of each phase^121^. Collectively, these modeling decisions shape tumor cell evolution post-treatment.

#### Deep learning models

In addressing the complexity of drug response prediction based on functionally interrelated molecular features, the field of machine learning (ML) is evolving to prioritize model interpretability alongside predictive accuracy. Interpretable deep learning models, such as the one introduced by Kuenzi et al., combined a visible neural network (VNN) with conventional artificial neural networks (ANN), which were trained on the responses of tumor cell lines to drugs^319^. This approach integrates tumor genetic profiles with drug structural information to enhance the prediction of therapeutic outcomes. The model, with an architecture similar to the hierarchy of biological systems, has aided in identifying subsystems important for drug response prediction. It highlights subsystems that align with established drug sensitivity mechanisms and unveils potential new pathways for investigation. These identified subsystems also offer potential avenues for synergistic drug combinations. Similarly, Zhang et al.’s deep learning model, which is constrained by signaling pathways, offers a structured and interpretable framework for drug response prediction and is capable of naturally integrating multi-omics data^320^. Gerdes et al. further contributed with an ensemble of ML models, which, by training on multi-omics data and cell responses to over 400 drugs, ranks drugs based on their anti-proliferative efficacy^321^. The model enhances predictive robustness and minimizes noise by utilizing internally normalized distance metrics of drug responses rather than individual features to generate predictions. These advancements signify a shift from opaque “black box” models to those embedding biological knowledge similar to the integration of prior knowledge found in the Bayesian method. This evolution deepens our understanding of how different molecular features interact to influence drug efficacy.

### Application of mathematical models in advancing cell cycle drug discovery

Cell cycle proteins are promising therapeutic targets because they are overexpressed or overactive in human cancers; some of them are required for tumorigenesis and are involved in cell cycle functions that modulate diverse aspects of cancer biology^322^. Several cell cycle inhibitors (e.g., CDK 4/6 inhibitors^323^, PLK1 inhibitor^324^, wee1 kinase inhibitor^325^, ATM inhibitor^326^) have emerged as potential therapeutic drugs for the treatment of cancers-both as single-agent therapy and in combination with traditional cytotoxic or molecular targeting agents. There are attempts by computational models to facilitate the identification of therapeutic targets by tapping into the extensive possibilities of the cell cycle.

There are different ways to discover the molecular targets affected by anticancer drugs. These include target identification and validation, high-throughput screening of chemical libraries for de novo drug discovery, or drug repositioning^327,328^. Drug repositioning, which aims to find new therapeutic indications for currently known and approved drugs, garners the attention of pharmaceutical researchers due to its high efficiency^329^. A variety of computational methods for drug repositioning have been proposed based on ML approaches^330,331^, network-based approaches^332,333^, and matrix decomposition approaches^334,335^.

Mechanistic modeling and ML approaches stand out in the quest for novel targets. ML presents a fresh outlook in expediting the identification of new druggable compounds through the exploration of drug design spaces^336^. With the rapid expansion of multi-omics data, the discovery of previously unknown cell cycle proteins^337^, and the discovery of cell cycle-regulated genes either experimentally^338^ or aided by deep learning-based approaches^339,340^ and classification algorithms^341^, ML emerges as a powerful analytical method to yield statistically valid predictions of cell cycle targeting drugs based on these multidimensional data sets. This capability to extract intricate nonlinear relationships between input data and output enables the prediction of potential target-disease causality^342^. The integration of diverse data types into ML models has proven to enhance the accuracy of these predictions^343^. For example, Jeon et al. built three support vector machine (SVM) classifiers with RBF kernel on integrated multi-omic and protein-protein interaction (PPI) data to predict proteins that could be potential drug targets for breast cancer (BrCa), pancreatic cancer (PaCa), and ovarian cancer (OvCa)^344^. Madhukar et al. developed a Bayesian ML approach named BANDIT, which integrates multiple data types, such as drug efficacies, post-treatment transcriptional responses, drug structures, reported adverse effects, bioassay results, and known targets. BANDIT generated around 4,000 previously unknown molecule-target predictions and identified 14 novel microtubule inhibitors^345^. Following target identification through these methods, target validation using ex vivo and in vivo models ensures the target’s physiological relevance before potential targets can enter clinical trials^346^.

Mechanistic models complement these discoveries by mapping functional dependencies among cell survival pathways on the network scale and encompass the essential components that have the potential to identify potential therapeutic targets. The application of PhysiBoSS by Ruscone et al. to simulate tumor cell invasion shows how these models can predict potential drug treatments and genetic perturbations capable of blocking or perturbing migration in cells that have already undergone EMT and started invading the surrounding ECM^347^. Similarly, Wang et al.’s 3D agent-based modeling identifies key therapeutic targets within the EGFR-TGF*β* signaling pathway in non-small cell lung cancer (NSCLC)^348^. Mombach et al. built a regulatory network of cell fate decisions upon DNA damage, focusing on the G1/S arrest network and the senescence regulatory pathway using a Boolean model^252^. The model predicts that CDC25A knockout enhances senescence, an outcome that could lead to a relevant target for cancer intervention. Poltz et al. also contributed to this field with a discrete logical model of the DNA damage repair (DDR) pathways, identifying sets of putative candidate target molecules to sensitize tumor cells to DNA-damaging therapeutics^349^. The integration of a pathway-derived mechanistic model with gene expression into ML methods represents a forward-thinking approach to identifying new drug targets. For instance, Esteban-Medina et al.’s exploration of the druggable space around the Fanconi anemia pathway, utilizing ML in tandem with mechanistic models, is a testament to the potential of this approach^350^.

### Leveraging cell cycle models to address drug resistance

Tumor cell resistance to anticancer drugs remains a significant challenge in cancer treatment. Multidrug resistance in tumor cells, where tumor cells become resistant to different agents with different functions, further complicates therapeutic outcomes^351^. This resistance arises from various mechanisms, including genetic mutations, overexpression of drug efflux pumps, epigenetic reprogramming, and activation of survival pathways that contribute to drug resistance (e.g., DNA repair pathway, apoptosis resistance pathway)^352–355^. Recent advances have led to novel strategies aimed at combating resistance, such as innovative drug formulations, combination therapies with different mechanisms of action, nanotechnology-based drugs, and modulation of the TME^356^. Despite progress, the knowledge gap in drug resistance persists, especially concerning differing resistance mechanisms among cancers harboring common driver mutations, TME’s role in favoring proliferation, and the impact of pre-existing and emerging genetic alterations during tumor evolution^357^. Cell cycle-mediated resistance (i.e., relative insensitivity to a chemotherapeutic agent because of the position of the cells in the cell cycle^358^) is increasingly apparent with our increased understanding of the impact cytotoxic agents have on the cell cycle. The impact of the cell cycle on drug sensitivity is experimentally shown in the literature^359–361^. Given the scope of this review, we concentrate on mechanistic models and data-driven models that are instrumental in developing effective strategies to combat drug resistance. Specifically, these models focus on multi-drug strategies or novel therapeutic agents designed to alleviate resistance, increase clinical benefit, and target cell cycle-specific vulnerabilities for improved therapeutic outcomes.

This intricate landscape of drug resistance is further complicated by intratumoral heterogeneity, a pivotal factor in advancing cancer treatment strategies. Intratumoral heterogeneity is characterized by three models: the clonal evolution model, highlighting how genetic variability and selective pressures give rise to cancer clones with growth advantage; the cancer stem-like cell (CSC) model, emphasizing a niche of self-renewing cells driving tumor growth; and cell plasticity highlighting the capacity of cancer cells to revert to a stem-like state^362^. Such heterogeneity, particularly cancer cell cycle heterogeneity, is recognized as a critical determinant of therapeutic resistance, complicating treatments targeting rapidly dividing cells^363^. The presence of resistant cells, whether pre-existing (intrinsic drug resistance) or emergent during treatment (acquired drug resistance), shifts the tumor milieu toward those resistant to therapy, often leading to recurrence after initial chemotherapy success^364^. The problem is exacerbated in tumors with a substantial fraction of dormant cells, which are notably difficult to target with conventional therapies aimed at dividing cells^365^. Various strategies, including direct eradication, maintaining dormancy, or inducing proliferative states to enhance drug sensitivity, have been explored, though their clinical efficacy remains to be proven^365^.

In response to these challenges, computational models have been developed to elucidate treatment-induced resistance evolution, tumor heterogeneity, and drug resistance mechanisms^366^. Research by Tzamali et al., Lorz et al., and Marcu et al. unveiled how treatment-induced selective pressures shape tumor heterogeneity and drug resistance^125,367,368^. Using ABM, PDE models, and probabilistic models, respectively, their studies provide insights that could inform innovative therapeutic strategies. Sun et al. developed an SDE to connect cellular mechanisms underlying cancer drug resistance to population-level patient survival, examining how targeted therapy-induced microenvironment adaptations contribute to resistance^369^. Schmitz et al. employed a cellular automata model to study treatment effects^370^. The simulation results reveal that the spatial distribution of treatment-resistant cells within heterogeneous tumors can variably influence patient survival times, and tumors exhibiting a range of mutation frequencies display distinct geometries. Further exploring the tumor microenvironment, Frieboes et al. delved into the roles of diffusion gradients and tissue compactness in drug resistance using the RD model^371^. Furthermore, Yang et al.’s multiscale agent-based model of glioblastoma integrated microenvironmental factors and genetic mutations to demonstrate how certain mutations accelerate phenotype heterogeneity and tumor invasion^372^. Their results show that angiogenesis proximal to tumors enhances tumor cell survival and drug resistance. These studies highlight the importance of strategies, such as those suggested by Saini et al., that aim to manipulate cell state transitions for optimized drug therapy amidst intratumoral heterogeneity^373^.

Understanding the interplay of cancer drug combinations with cell cycle phases is pivotal for developing effective combination therapies. This knowledge could guide the selection of drug combinations that work synergistically, an approach increasingly crucial for advancing cancer therapy^282^. Insights from modeling efforts^374,375^ suggest that the cell cycle-mediated drug resistance, due to some cancer cells being in untargeted phases, may potentially be countered by administering multiple doses of phase-specific chemotherapy targeting cells across all phases with careful sequencing and scheduling, a concept supported by preclinical studies and showing promise in clinical trials. Furthermore, model simulations indicate targeting both cell cycle vulnerabilities and differentiation pathways can yield a synergistic effect^376^. The success of combination therapy depends on several factors. Modeling studies have investigated the effect of stress-activated signaling^377^, cell turnover^378^, and stochastic noise that inherently exists in signal transduction and phenotypic regulation^379^ on apoptosis induced by combination therapy. Metronomic chemotherapy, which involves smaller and more frequent doses to thwart rapid cell proliferation and maintain tumor control, emerges as a strategic response to the risks of treatment lapses and tumor recurrence from single-dose treatments that spare resistant cells^262^. Mathematical models also optimize intermittent therapy that curtails the evolution of drug resistance and allows recovery periods between treatments to mitigate side effects^380–382^. The combination of conventional chemotherapy and other modalities, such as radiation, targeted therapy, and gene therapy to combat resistance, are studied in mathematical models as reviewed by Sun et al.^354^.

### Model-driven approaches for dose optimization

Facing challenges like resistance and adverse impacts on the immune system from maximum tolerated dose (MTD) strategies, oncology is shifting toward dose optimization for better patient outcomes^383–385^. This transition benefits from the integration of QSP models and PK/PD models, coupled with optimal control theory (OCT) to devise individualized therapies efficiently^386^.

OCT offers a rigorous approach to dose optimization^387,388^. It emphasizes an optimization framework to identify the treatment regimens that best meet the performance measure of a clinical goal subject to specific constraints. For instance, when the endpoint is therapeutic efficacy, the optimization scheme seeks to minimize the number of cancer cells or tumor volume under the constraints of treatment costs like toxicity and gained resistance. The numerical solutions to optimal control problems can be found either indirectly, by formulating necessary optimality conditions that the optimal control trajectory must satisfy^389,390^, or directly through discretization of the state and control variables followed by iterative optimization techniques, bypassing the need for an analytical expression of optimality conditions.

Cell cycle dynamics play a pivotal role in determining optimal drug dosages, given the cycle dependency of various cancer treatments, such as radiotherapy, chemotherapies, and therapies targeting cell cycle proteins. To study this, mathematical models, both discrete and continuous, depict tumors as a large population of cells, allowing for considering multiple subpopulations with distinct characteristics. However, a notable limitation of these models is their reliance on predefined cell type diversity, such as cells in different cell cycle phases or those categorized by specific phenotypes like proliferating, quiescent, hypoxic, or necrotic states. This approach does not allow for the emergence of new subpopulations during simulations, potentially oversimplifying the complex nature of tumor evolution. Despite this, the ability to track changes in cell counts across different types under varied treatment schedules provides invaluable insights, informing the optimization of treatment plans.

Chronotherapy, which aligns cancer treatment with the body’s endogenous circadian rhythms, has been shown experimentally to improve anticancer treatment outcomes^391,392^. The mathematical model can help leverage chronotherapy to predict the optimal treatment time. In^212^, Billy et al. applied an age-structured model to numerically solve a cancer chronotherapeutic optimization problem. The transition kernel for the circadian control free cell population was modified to integrate factors like natural circadian control and drug infusion effects. This could lead to an infusion strategy that minimizes tumor growth. Hesse et al. combined a core-lock model and an irinotecan chronoPK-PD model in their study^393^, enabling cytotoxicity timing predictions for irinotecan using circadian gene expression data and irinotecan metabolism-related mRNAs.

In the field of dose optimization, diverse modeling frameworks are employed to study the myriad factors influencing treatment efficacy, each presenting unique benefits and challenges. ODE models have been effective in optimizing dosing schedules for cell cycle-specific chemotherapies^312,394–398^. Meanwhile, the integration of OCT with a linear quadratic model refines radiotherapy fractionation protocols^399^, and age-structured models provide a foundation for deriving customized treatment procedures^400^. The spatial arrangement of cells, influenced by variations in treatment sensitivity and factors like TME and cell cycle differences, is crucial in determining treatment outcomes. As such, spatially distributed models, whether continuous^401,402^ or discrete agent-based models^403^, address the spatial complexities of tumor cell distributions and the evolving TME despite the computational intensity required to manage spatial information.

Personalized medicine greatly benefits from dose optimization as it ensures treatments are tailored to the tumor’s particular expression profile. Yet, integrating patient-specific mathematical models with optimal control theory can be challenging due to factors such as limited availability of clinical data, difficulty in developing multimodal data fusion methods, and challenges in identifying the biomarkers determining the treatment outcome variability. Additionally, applying preclinical treatment schedules to clinical settings can be complex. Despite these hurdles, the potential for breakthroughs in personalized medicine is immense. Quantitative imaging data can now more accurately profile individual tumors, while multiscale modeling further enhances the predictive accuracy of treatment outcomes. To ensure a smoother transition from preclinical findings to clinical implementations, strategies such as parameter adjustment from mouse to human or employing allometric scaling for physiological differences are helpful^404^. In an effort to use computational models to conduct virtual experiments and sub-group analyses, the concepts of digital twins and virtual patients (VP) have been introduced. VPs are constructed from aggregated patient data and made possible by QSP models by parameter adjustments, thereby providing a realistic yet anonymized disease overview. They are ideal for creating virtual control groups in drug evaluation studies of predictable diseases like advanced cancers^405^. Digital twins are virtual representations of a patient or a disease. It requires advanced computing and biotechnologies to create dynamic in silico models that accurately mirror a patient’s condition over time and across treatments^406,407^. Both VPs and digital twins can be seamlessly integrated into medical workflows to enhance treatment decision-making. These tools improve the accuracy of pre-treatment response predictions and recurrence risk assessments, and they also facilitate the design of clinical trials^406–408^.

We summarized all the models reviewed in Sections ''Applications of cell cycle models to model biological phenomena'' and ''Enhancing cancer treatment strategies through computational modeling of the cell cycle'' in Supplementary Table 1.

## Discussion

Computational modeling has emerged as an indispensable tool in the study of the cell cycle and its implications for cancer treatment strategies. Through the integration of diverse modeling approaches, ranging from deterministic and stochastic models to multiscale and agent-based frameworks, researchers have gained unparalleled insights into the cell cycle’s intricate dynamics. These models offer a unique vantage point from which to explore the multifaceted interactions within the TME, the regulatory networks governing cell proliferation and death, and the mechanisms of drug resistance and therapeutic intervention. Crucially, the adaptability of mathematical and computational models enables their ongoing evolution, ensuring they remain at the forefront of scientific inquiry into cellular processes. By capturing the complex behaviors of tumor cells and their interactions with the TME, these models serve not only as powerful predictive tools but also as platforms for hypothesis testing and the refinement of treatment strategies. Looking forward, the integration of computational models with experimental and clinical data promises to enhance our understanding of cancer biology and to inform the development of more effective, personalized treatment regimens. As these models are refined and their capabilities expanded, their utility in guiding clinical decisions and in identifying new therapeutic targets is expected to increase, contributing to advances in precision oncology.

## Supplementary information

Supplementary Information
