# Author Correction: A comprehensive review of computational cell cycle models in guiding cancer treatment strategies

**DOI:** 10.1038/s41540-026-00837-6

**Published:** 2026-10-08

**Authors:** Chenhui Ma, Evren Gurkan-Cavusoglu

**Affiliations:** Department of Electrical, Computer and Systems Engineering, Case Western Reserve University, Cleveland, OH USA

**Keywords:** Software, Computational science

Correction to: *npj Systems Biology and Applications* https://doi.org/10.1038/s41540-024-00397-7, published online 05 July 2024

In the Abstract section, the word ‘viability’ should have read ‘variability’. The original article has been corrected.

