# Supplementary material for: A comprehensive review of computational cell cycle models in guiding cancer treatment strategies
Source: NPJ Syst Biol Appl. 2024 Jul 5;10:71. doi: 10.1038/s41540-024-00397-7 (PMC11226463; doi:10.1038/s41540-024-00397-7)
Supplement: Supplementary file 1 — Supplementary Information [file 41540_2024_397_MOESM1_ESM.pdf]

Supplementary table for a comprehensive review of computational cell cycle models in guiding cancer treatment strategies

Supplementary Table 1: Computational models that are discussed in Sections 4 and 5.

4.1. Modeling cell cycle reaction network centering on cyclin/CDK complexes

Study	Ref	Modeling techniques
Self-organization of the cyclin/CDK network driving the mammalian cell cycle	Gerard et al. 2009 [1]	ODE model
Regulation of cell cycle arrest and proliferation by ECM and contact inhibition	Gerard et al. 2014 [2]	ODE model
Feedback loops' role in maintaining CDK oscillations' robustness against molecular noise	Gerard et al. 2011, 2012 [3, 4]	ODE model
Cell division and cell growth	Yang et al. 2006 [5]	PDE
Mammalian cell cycle regulation	Weis et al. 2014 [6]	ODE
Wee1 and Cdc25's reversed roles in stabilizing G2 phase and enhancing cell cycle checkpoint	Hernansaiz-Ballesteros et al. 2021 [7]	ODE
Roles for feedback regulation in limiting cellular variability	Barik et al. 2016 [8]	Stochastic model
Cell cycle regulation	Laomettachit et al. 2016 [9]	Hybrid model
Cell cycle regulation	Mura et al. 2008 [10]	Stochastic Petri Nets
CDK oscillations and cellular heterogeneity	Gerard et al. 2019 [11]	Stochastic model
Yeast cell cycle protein interaction network	Zhang et al. 2006 [12]	Boolean model
Cell cycle sequence of fission yeast	Davidich et al. 2008 [13]	Boolean model

Table 1 continued from previous page

Conditionally stable circuits driving the cell cycle	Deritei et al. 2019 [14]	Boolean model
Mammalian cell cycle regulation	Singhania et al. 2011 [15]	Hybrid model

4.2. Models to study cell cycle variability

Study	Ref	Modeling techniques
Variability and stochastic nature of cell cycle progression	Smith et al. 1973 [16]	Probabilistic model
Random transitions in mammalian cell cycles	Brooks et al. 1980 [17]	Probabilistic model
Hippocampal neurogenesis	Li et al. 2017 [18]	Branching process
Proliferation and differentiation of O-2A progenitor cells	Hyrien et al. 2005 [19]	Branching process
Generational inheritance in cell proliferation	Stivers et al. 1996 [20]	Branching process
Regulated generation of oligodendrocytes in cell culture	Boucher et al. 1999 [21]	Branching process
Saddlepoint approximations to the moments of branching processes	Hyrien et al. 2010 [22]	Branching process
Analysis of CFSE-labeling experiments	Hyrien et al. 2010 [23]	Branching process
Cell differentiation	Nordon et al. 2011 [24]	Branching process
Cell proliferation	Yates et al. 2017 [25]	Multi-stage model
Birth and death processes	Belluccini et al. 2022 [26]	Multi-stage model
Normally-hidden inherent synchronization in cell proliferation	Vittadello et al. 2019 [27]	Multi-stage model
Effect of cell cycle variability on messenger RNA abundance	Perez-Carrasco et al. 2020 [28]	Multi-stage model

4.4. Cell size control

Study	Ref	Modeling techniques
Molecular noise and size control in the budding yeast cell cycle	Talia et al. 2007 [29]	Stochastic model
Size control in the budding yeast cell cycle	Ahmadian et al. 2019 [30]	Stochastic model

Table 1 continued from previous page

Cell cycle distributions in synchrony experiments	Orlando et al. 2007 [31]	Probabilistic model
Single-cell stochasticity, size control in asymmetrically dividing cells	Barber et al. 2021 [32]	Probabilistic model
Cell size control	Novak et al. 2001, Li et al. 2010, Facchetti et al. 2019 [33–35]	ODE model
Cell size control	Hannsgen et al. 1985, Tyson et al. 1968 [36, 37]	Probabilistic model

4.5. Stem cell renewal dynamics

Study	Ref	Modeling techniques
Stem cell regulation	Marciniak-Czochra et al. 2009, Bessonov et al. 2019 [38, 39]	ODE model
Symmetric and asymmetric stem cell division	Yan et al. 2023 [40]	RD model
Adult neurogenesis, cell cycle progression and differentiation	Stopka et al. 2019 [41]	Stochastic model

4.6. Interplay between DNA replication and cell cycle progression

Study	Ref	Modeling techniques
Link between molecular-scale events and single-cell characteristics assessed by multiparameter cytometry	Li et al. 2014 [42]	Stochastic model
DNA replication, 3D protein mobility dynamics	Windhager et al. 2022 [43]	Stochastic hybrid model
DNA replication across a complete genome	Lygeros et al. 2008 [44]	Stochastic hybrid model
DNA replication	Koutroumpas et al. 2011 [45]	Stochastic hybrid model
DNA replication timing in human cells.	Gindin et al. 2014 [46]	Stochastic model
Modeling the mammalian cell cycle in an autonomous manner, mechanisms to prevent rereplication are included	Williams et al. 2023 [47]	ODE model

4.7.DNA Damage repair pathway and cell cycle progression

Table 1 continued from previous page

Study	Ref	Modeling techniques
Cell cycle progression and DNA double-strand breaks (DSBs) repair post-irradiation	Mohseni-Salehi et al. 2020 [48]	Stochastic model
p53/Mdm2 oscillation system and G2/M phase in the cell cycle	Tashima et al. 2007 [49]	ODE model
Structured dynamics of the cell cycle at multiple scales.	Hodgkinson et al. 2023 [50]	PDE model
DNA damage repair and cell response in relation to p53 system exposed to ionizing radiation	Hu et al. 2022 [51]	Hybrid model
Onset of senescence at the G1/S cell cycle checkpoint	Mombach et al. 2014 [52]	Boolean model

4.8. Models to explore the crosstalk between cell cycle network and signaling pathways in carcinogenesis

Study	Ref	Modeling techniques
Influence of MAPK network on cancer cell fate decision	Grieco et al. 2013 [53]	Boolean model
Aberrant cell cycle progression driven by hyperactive PI3K	Sizek et al. 2019 [54]	Boolean model
A scalable, open-source implementation of a large-scale mechanistic model for single cell proliferation and death signaling	Erdem et al. 2022 [55]	Hybrid model

4.9. Impact of hypoxia, nutrient deficiency, angiogenesis, mechanical forces in TME on tumor growth and cell cycle dynamics

Study	Ref	Modeling techniques
Cell cycle progression under cyclic hypoxia	Celora et al. 2022 [56]	PDE model
The effects of cell-cycle heterogeneity on the response of a solid tumor to chemotherapy	Powathil et al. 2012 [57]	Hybrid multiscale model
The function of Warburg effect in TME	Shamsi et al. 2018 [58]	Cellular automaton model
Avascular tumor growth	Jiang et al. 2005 [59]	Multiscale model
Cancer growth evolution and invasion	Anderson et al. 2005 [60]	Multiscale model

Table 1 continued from previous page

Cancer growth using features derived from microCT images	Zangooei et al. 2021 [61]	Multiscale model
Angiogenesis and vascular tumor growth	Owen et al. 2009 [62]	Multiscale model
Progression of ductal carcinoma in situ	Macklin et al. 2012 [63]	ABM
Cerebral tumor growth	Elazab et al. 2019 [64]	RD model

4.10. Mathematical models to study tumor-immune dynamics within the TME and immunotherapy outcomes

Study	Ref	Modeling techniques
Tumor growth and response to immunotherapy	Ruiz-Martinez et al. 2022 [65]	spQSP model
Personalized prediction of triple-negative breast cancer immunotherapy response	Zhang et al. 2021 [66]	spQSP model
Intratumoral heterogeneity and immunoarchitecture	Nikfar et al. 2023 [67]	spQSP model
Bladder cancer immune landscape alteration through dysregulated FGFR3 signaling	Bergman et al. 2024 [68]	ABM
Immunotherapy and stroma-targeting therapies in human colorectal cancer	Kather et al. 2017 [69]	ABM

5.2 Application of computational models to quantify efficacy of treatment targeting cell cycle

Study	Ref	Modeling techniques
Cell cycle progression in the MCF-7 breast cancer cell line	Simms et al. 2012 [70]	ODE model
Mammalian cell cycle control system in the presence of DNA damage stress	Abroudi et al. 2017 [71]	ODE model
Prediction of the cellular responses to chemotherapy	Alkan et al. 2018 [72]	ODE model
Drug-induced cell cycle arrest, cell-to-cell variation during mitosis	Bae et al. 2019 [73]	ODE model
Human tumor cell populations both unperturbed and exposed to a range of cancer therapies	Basse et al. 2007 [74]	Age-structured model
The effect of the G1-S transition checkpoint	Chaffey et al. 2014 [75]	Age-structured model
Cell cycle progression based on cyclin-dependent kinases activity	Pisu et al. 2015 [76]	PDE model

Table 1 continued from previous page

Radiation-induced cell cycle perturbation	Lonati et al. 2021 [77]	DNA-structured model
Heterogeneous cell response to anticancer treatments.	Falcetta et al. 2013 [78]	Discrete PDE
Antitumor activity of LY2835219, in mice bearing human tumor xenografts	Tate et al. 2014 [79]	Mechanistic PK/PD
Antitumor activity of 5-Fluorouracil (5-FU)	Ma et al. 2022 [80]	Mechanistic PK/PD
Cell cycle effects for gemcitabine and trabectedin combinations in pancreatic cancer cells	Miao et al. 2016 [81]	Mechanistic PK/PD
Combined treatment of gemcitabine and birinapant in pancreatic cancer cells	Zhu et al. 2015 [82]	Mechanistic PK/PD
Response to cyclotherapy	Jackson et al. 2017 [83]	Mechanistic PK/PD
Invasive solid tumor growth in heterogeneous microenvironment under chemotherapy	Xie et al. 2018 [84]	Hybrid ABM
The interplay between chemokine receptor CCR5 expression, cancer stem cells, and hypoxia	Norton et al. 2017 [85]	ABM
Interaction between crowding and growth in tumors with stem cells	Meacci et al. 2023 [86]	Cellular automata model
Checkpoint-oriented cell cycle simulation	Bernard et al. 2019 [87]	ABM
The effect of iododeoxyuridine and ionizing radiation on cell cycle dynamics in colorectal cancer cells	Gurkan et al. 2007, 2013 [88, 89]	Probabilistic model
Prediction of drug response and synergy in human cancer cells	Kuenzi et al. 2020 [90]	Deep learning model
Prediction of anticancer drug response with constraints of signaling pathway	Zhang et al. 2021 [91]	Deep learning model
Prediction the efficacy of anti-cancer drugs	Gerdes et al. 2021 [92]	ML

5.3 Application of mathematical models in advancing cell cycle drug discovery

Study	Ref	Modeling techniques
Identification of novel cancer drug targets	Jeon et al. 2014 [93]	ML
Drug target identification using diverse data types	Madhukar et al. 2019 [94]	Bayesian ML
Cancer cell invasion	Ruscone et al. 2023 [95]	Multiscale model
Identification of therapeutic targets in a combined EGFR–TGFβR signalling cascade	Wang et al. 2012 [96]	ABM

Table 1 continued from previous page

Dynamics of p53 and $NF-\kappa B$ regulation in response to DNA damage, identification of target proteins	Poltz et al. 2012 [97]	Boolean model
Exploration of the druggable space around the Fanconi anemia pathway	Esteban-Medina et al. 2019 [98]	ML and mechanistic model

5.4 Leveraging mathematical cell cycle models to address drug resistance

Study	Ref	Modeling techniques
Impact of cell cycle length heterogeneity on cancer cell growth dynamics and treatment response	Tzamali et al. 2020 [99]	ABM
Populational adaptive evolution, chemotherapeutic resistance, and multiple anti-cancer therapies	Lorz et al. 2013 [100]	PDE model
Tumor resistance to cisplatin	Marcu et al. 2005 [101]	Probabilistic model
Therapy-induced cancer drug resistance, population survival rates	Sun et al. 2016 [102]	Stochastic model
Brain tumor treatment and resistance	Schmitz et al. 2002 [103]	Cellular automata model
Prediction of drug response	Frieboes et al. 2009 [104]	PDE model
Drug resistance in glioblastoma with gene mutations and angiogenesis	Yang et al. 2023 [105]	ABM
The development of chemotherapeutic drug resistance in cancer	Powathil et al. 2014 [106]	Hybrid multiscale model
Chemotherapeutic drug resistance	Hamis et al. 2018 [107]	Hybrid multiscale model
Glioma differentiation signaling pathways, drug combination therapy	Sun et al. 2015 [108]	ODE model
Drug resistance in cancer	Komarova et al. 2006 [109]	Stochastic model
The relationship between stochastic noise and drug efficacy in differentiation therapy	Sun et al. 2016 [110]	Stochastic model
The effects of cell cycle heterogeneity on the response of a solid tumor to chemotherapy	Powathil et al. 2012 [57]	Hybrid multiscale model
The transitions between endocrine therapy responsive and resistant states in breast cancer	Chen et al. 2014 [111]	Stochastic model
Response of prostate cancer under intermittent androgen suppression	Hirata et al. 2012 [112]	ODE model

Table 1 continued from previous page

Prostate cancer progression in response to androgen ablation therapy	Jain et al. 2011 [113]	ODE model

5.5 Model-driven approaches for dose optimization

Study	Ref	Modeling techniques
Synchronization and control of proliferation in cycling cells	Billy et al. 2014 [114]	Age-structured model
Circadian-based timing optimization for irinotecan in colorectal cancer	Hesse et al. 2021 [115]	ODE model
Optimal control in cell-cycle-specific cancer therapy to avoid overdestruction of the bone marrow	Panetta et al. 2006 [116]	ODE model
Optimal strategy derivation for tumor–immune dynamics and chemotherapy	de Pillis et al. 2007 [117]	ODE model
Chemotherapy scheduling to minimize tumor size with drug resistance and toxicity considerations	Dua et al. 2008 [118]	ODE model
Multi-objective multi-drug scheduling schemes for cell cycle-specific cancer treatment	Alam et al. 2013 [119]	ODE model
Combined treatment of gemcitabine and birinapant in pancreatic cancer cells	Zhu et al. 2015 [82]	ODE model
Optimal treatment strategy for tumor model under immune suppression	Kim et al. 2014 [120]	ODE model
Optimized radiation dosing schedules for PDGF-driven glioblastoma	Leder et al. 2014 [121]	Linear quadratic model
Cell population dynamics, drug delivery optimization	Clairambault et al. 2016 [122]	Age-structured model
Scheduling cancer treatment with a combination of VEGF inhibitor and chemotherapy drugs	Lai et al. 2019 [123]	RD model
Optimization of antitumor radiotherapy fractionation	Kuznetsov et al. 2023 [124]	PDE model
Solid tumor treatment response prediction to chemotherapy and radiotherapy	Powathil et al. 2013 [125]	Hybrid multiscale model

References

[1] Gérard, C., Goldbeter, A.: Temporal self-organization of the cyclin/Cdk network driving the mammalian cell cycle. Proc Natl Acad Sci U.S.A **106**(51), 21643–21648 (2009) https://doi.org/10.1073/pnas.0903827106

[2] Gérard, C., Goldbeter, A.: The balance between cell cycle arrest and cell proliferation: control by the extracellular matrix and by contact inhibition. Interface Focus **4**(3), 20130075 (2014) https://doi.org/10.1098/rsfs.2013.0075

[3] Gérard, C., Goldbeter, A.: A skeleton model for the network of cyclin-dependent kinases driving the mammalian cell cycle. Interface Focus **1**(1), 24 (2011) https://doi.org/10.1098/rsfs.2010.0008

[4] Gérard, C., Gonze, D., Goldbeter, A.: Effect of positive feedback loops on the robustness of oscillations in the network of cyclin-dependent kinases driving the mammalian cell cycle. FEBS J **279**(18), 3411–3431 (2012) https://doi.org/10.1111/j.1742-4658.2012.08585.x

[5] Yang, L., Han, Z., MacLellan, W.R., Weiss, J.N., Qu, Z.: Linking Cell Division to Cell Growth in a Spatiotemporal Model of The Cell Cycle. J Theor Biol **241**(1), 120 (2006) https://doi.org/10.1016/j.jtbi.2005.11.020

[6] Weis, M.C., Avva, J., Jacobberger, J.W., Sreenath, S.N.: A Data-Driven, Mathematical Model of Mammalian Cell Cycle Regulation. PLoS One **9**(5), 97130 (2014) https://doi.org/10.1371/journal.pone.0097130

[7] Hernansaiz-Ballesteros, R.D., Földi, C., Cardelli, L., Nagy, L.G., Csikász-Nagy, A.: Evolution of opposing regulatory interactions underlies the emergence of eukaryotic cell cycle checkpoints. Sci Rep **11**(11122), 1–10 (2021) https://doi.org/10.1038/s41598-021-90384-3

[8] Barik, D., Ball, D.A., Peccoud, J., Tyson, J.J.: A Stochastic Model of the Yeast Cell Cycle Reveals Roles for Feedback Regulation in Limiting Cellular Variability. PLoS Comput Biol **12**(12), 1005230 (2016) https://doi.org/10.1371/journal.pcbi.1005230

[9] Laomettachit, T., Chen, K.C., Baumann, W.T., Tyson, J.J.: A Model of Yeast Cell-Cycle Regulation Based on a Standard Component Modeling Strategy for Protein Regulatory Networks. PLoS One **11**(5), 0153738 (2016) https://doi.org/10.1371/journal.pone.0153738

[10] Mura, I., Csikász-Nagy, A.: Stochastic Petri Net extension of a yeast cell cycle model. J Theor Biol **254**(4), 850–860 (2008) https://doi.org/10.1016/j.jtbi.2008.07.019

[11] Gérard, C., Gonze, D., Goldbeter, A.: Revisiting a skeleton model for the mammalian cell cycle: From bistability to Cdk oscillations and cellular heterogeneity. J Theor Biol **461**, 276–290 (2019) https://doi.org/10.1016/j.jtbi.2018.10.042

[12] Zhang, Y., *et al.*: Stochastic model of yeast cell-cycle network. Physica D **219**(1), 35–39 (2006) https://doi.org/10.1016/j.physd.2006.05.009

[13] Davidich, M.I., Bornholdt, S.: Boolean Network Model Predicts Cell Cycle Sequence of Fission Yeast. PLoS One **3**(2), 1672 (2008) https://doi.org/10.1371/journal.pone.0001672

[14] Deritei, D., Rozum, J., Ravasz Regan, E., Albert, R.: A feedback loop of conditionally stable circuits drives the cell cycle from checkpoint to checkpoint. Sci Rep **9**(16430), 1–19 (2019) https://doi.org/10.1038/s41598-019-52725-1

[15] Singhania, R., Sramkoski, R.M., Jacobberger, J.W., Tyson, J.J.: A Hybrid Model of Mammalian Cell Cycle Regulation. PLoS Comput Biol **7**(2), 1001077 (2011) https://doi.org/10.1371/journal.pcbi.1001077

[16] Smith, J.A., Martin, L.: Do cells cycle? Proc Natl Acad Sci U.S.A **70**(4), 1263–1267 (1973) https://doi.org/10.1073/pnas.70.4.1263

[17] Brooks, R.F., Bennett, D.C., Smith, J.A.: Mammalian cell cycles need two random transitions. Cell **19**(2), 493–504 (1980) https://doi.org/10.1016/0092-8674(80)90524-3

[18] Li, B., *et al.*: Multitype Bellman-Harris branching model provides biological predictors of early stages of adult hippocampal neurogenesis. BMC Syst. Biol. **11**(5), 1–16 (2017) https://doi.org/10.1186/s12918-017-0468-3

[19] Hyrien, O., Mayer-Pröschel, M., Noble, M., Yakovlev, A.: A Stochastic Model to Analyze Clonal Data on Multi-Type Cell Populations. Biometrics **61**(1), 199–207 (2005) https://doi.org/10.1111/j.0006-341X.2005.031210.x

[20] Stivers, D.N., Kimmel, M., Axelrod, D.E.: A discrete-time, multi-type generational inheritance branching process model of cell proliferation. Math Biosci **137**(1), 25–50 (1996) https://doi.org/10.1016/S0025-5564(96)00066-1

[21] Boucher, K., Y. Yakovlev, A., Mayer-Proschel, M., Noble, M.: A stochastic model of temporally regulated generation of oligodendrocytes in cell culture. Math Biosci **159**(1), 47–78 (1999) https://doi.org/10.1016/S0025-5564(99)00010-3

[22] Hyrien, O., Chen, R., Mayer-Pröschel, M., Noble, M.: Saddlepoint approximations to the moments of multitype age-dependent branching processes, with applications. Biometrics **66**(2), 567–577 (2010) https://doi.org/10.1111/j.1541-0420.2009.01281.x

[23] Hyrien, O., Chen, R., Zand, M.S.: An age-dependent branching process model for the analysis of CFSE-labeling experiments. Biol. Direct **5**(1), 1–17 (2010) https://doi.org/10.1186/1745-6150-5-41

[24] Nordon, R.E., Ko, K.-H., Odell, R., Schroeder, T.: Multi-type branching models to describe cell differentiation programs. J. Theor. Biol. **277**(1), 7–18 (2011) https://doi.org/10.1016/j.jtbi.2011.02.006

[25] Yates, C.A., Ford, M.J., Mort, R.L.: A Multi-stage Representation of Cell Proliferation as a Markov Process. Bull Math Biol **79**(12), 2905 (2017) https://doi.org/10.1007/s11538-017-0356-4

[26] Belluccini, G., López-García, M., Lythe, G., Molina-París, C.: Counting generations in birth and death processes with competing Erlang and exponential waiting times. Sci Rep **12**(11289), 1–20 (2022) https://doi.org/10.1038/s41598-022-14202-0

[27] Vittadello, S.T., McCue, S.W., Gunasingh, G., Haass, N.K., Simpson, M.J.: Mathematical models incorporating a multi-stage cell cycle replicate normally-hidden inherent synchronization in cell proliferation. J R Soc Interface **16**(157) (2019) https://doi.org/10.1098/rsif.2019.0382

[28] Perez-Carrasco, R., Beentjes, C., Grima, R.: Effects of cell cycle variability on lineage and population measurements of messenger RNA abundance. J. R. Soc. Interface **17**(168) (2020) https://doi.org/10.1098/rsif.2020.0360

[29] Talia, S.D., Skotheim, J.M., Bean, J.M., Siggia, E.D., Cross, F.R.: The effects of molecular noise and size control on variability in the budding yeast cell cycle. Nature **448**, 947–951 (2007) https://doi.org/10.1038/nature06072

[30] Ahmadian, M., Tyson, J.J., Cao, Y.: A stochastic model of size control in the budding yeast cell cycle. BMC Bioinf **20**(12), 1–13 (2019) https://doi.org/10.1186/s12859-019-2839-9

[31] Orlando, D.A., *et al.*: A probabilistic model for cell cycle distributions in synchrony experiments. Cell Cycle **6**(4), 478–488 (2007) https://doi.org/10.4161/cc.6.4.3859

[32] Barber, F., Min, J., Murray, A.W., Amir, A.: Modeling the impact of single-cell stochasticity and size control on the population growth rate in asymmetrically dividing cells. PLoS Comput Biol **17**(6), 1009080 (2021) https://doi.org/10.1371/journal.pcbi.1009080

[33] Novak, B., Pataki, Z., Ciliberto, A., Tyson, J.J.: Mathematical model of the cell division cycle of fission yeast. Chaos **11**(1), 277–286 (2001) https://doi.org/10.1063/1.1345725

[34] Li, B., Shao, B., Yu, C., Ouyang, Q., Wang, H.: A mathematical model for cell size control in fission yeast. J Theor Biol **264**(3), 771–781 (2010) https://doi.org/10.1016/j.jtbi.2010.03.023

[35] Facchetti, G., Knapp, B., Flor-Parra, I., Chang, F., Howard, M.: Reprogramming Cdr2-Dependent Geometry-Based Cell Size Control in Fission Yeast. Curr Biol **29**(2), 350 (2019) https://doi.org/10.1016/j.cub.2018.12.017

[36] Hannsgen, K.B., Tyson, J.J.: Stability of the steady-state size distribution in a model of cell growth and division. J Math Biol **22**(3), 293–301 (1985) https://doi.org/10.1007/BF00276487

[37] Tyson, J.J., Diekmann, O.: Sloppy size control of the cell division cycle. J Theor Biol **118**(4), 405–426 (1986) https://doi.org/10.1016/S0022-5193(86)80162-X

[38] Marciniak-Czochra, A., Stiehl, T., Ho, A.D., Jäger, W., Wagner, W.: Modeling of asymmetric cell division in hematopoietic stem cells—regulation of self-renewal is essential for efficient repopulation. Stem Cells and Development **18**(3), 377–386 (2009) https://doi.org/10.1089/scd.2008.0143

[39] Bessonov, N., Pinna, G., Minarsky, A., Harel-Bellan, A., Morozova, N.: Mathematical modeling reveals the factors involved in the phenomena of cancer stem cells stabilization. PLoS One **14**(11), 0224787 (2019) https://doi.org/10.1371/journal.pone.0224787

[40] Yan, K., Wang, M., Qiu, Z., Xu, M.: A cell model about symmetric and asymmetric stem cell division. J Theor Biol **560**, 111380 (2023) https://doi.org/10.1016/j.jtbi.2022.111380

[41] Stopka, A., Boareto, M.: A stochastic model of adult neurogenesis coupling cell cycle progression and differentiation. J Theor Biol **475**, 60–72 (2019) https://doi.org/10.1016/j.jtbi.2019.05.014

[42] Li, B., *et al.*: Different rates of DNA replication at early versus late S-phase sections: Multiscale modeling of stochastic events related to DNA content/EdU (5-ethynyl-2′deoxyuridine) incorporation distributions. Cytometry A **85**(9), 785–797 (2014) https://doi.org/10.1002/cyto.a.22484

[43] Windhager, J., et al.: A stochastic hybrid model of DNA replication incorporating 3D protein mobility dynamics. bioRxiv, 583187 (2022) https://doi.org/10.1101/583187

[44] Lygeros, J., *et al.*: Stochastic hybrid modeling of DNA replication across a complete genome. Proc Natl Acad Sci U.S.A **105**(34), 12295–12300 (2008) https://doi.org/10.1073/pnas.0805549105

[45] Koutroumpas, K., Lygeros, J.: Modeling and analysis of DNA replication. Automatica **47**(6), 1156–1164 (2011) https://doi.org/10.1016/j.automatica.2011.02.007

[46] Gindin, Y., Valenzuela, M.S., Aladjem, M.I., Meltzer, P.S., Bilke, S.: A chromatin structure-based model accurately predicts DNA replication timing in human cells. Mol Syst Biol (2014) https://doi.org/10.1002/msb.134859

[47] Williams, K.S., Secomb, T.W., El-Kareh, A.W.: An autonomous mathematical

model for the mammalian cell cycle. J Theor Biol **569**, 111533 (2023) https://doi.org/10.1016/j.jtbi.2023.111533

[48] Mohseni-Salehi, F.S., Zare-Mirakabad, F., Sadeghi, M., Ghafouri-Fard, S.: A Stochastic Model of DNA Double-Strand Breaks Repair Throughout the Cell Cycle. Bull Math Biol **82**(1), 1–36 (2020) https://doi.org/10.1007/s11538-019-00692-z

[49] Tashima, Y., *et al.*: Mathematical modeling of G2/M phase in the cell cycle with involving the p53/Mdm2 oscillation system. In: World Congress on Medical Physics and Biomedical Engineering 2006, pp. 197–200. Springer, Berlin, Germany (2007). https://doi.org/10.1007/978-3-540-36841-0_58

[50] Hodgkinson, A., Tursynkozha, A., Trucu, D.: Structured dynamics of the cell-cycle at multiple scales. Front Appl Math Stat **9**, 1090753 (2023) https://doi.org/10.3389/fams.2023.1090753

[51] Hu, A., et al.: Modeling of DNA Damage Repair and Cell Response in Relation to p53 System Exposed to Ionizing Radiation. Int J Mol Sci **23**(19) (2022) https://doi.org/10.3390/ijms231911323

[52] Mombach, J.C.M., Bugs, C.A., Chaouiya, C.: Modelling the onset of senescence at the G1/S cell cycle checkpoint. BMC Genomics **15**(7), 1–11 (2014) https://doi.org/10.1186/1471-2164-15-S7-S7

[53] Grieco, L., Calzone, L., Bernard-Pierrot, I., Radvanyi, F., Kahn-Perlès, B., Thieffry, D.: Integrative Modelling of the Influence of MAPK Network on Cancer Cell Fate Decision. PLoS Comput Biol **9**(10), 1003286 (2013) https://doi.org/10.1371/journal.pcbi.1003286

[54] Sizek, H., Hamel, A., Deritei, D., Campbell, S., Regan, E.R.: Boolean model of growth signaling, cell cycle and apoptosis predicts the molecular mechanism of aberrant cell cycle progression driven by hyperactive PI3K. PLoS Comput Biol **15**(3), 1006402 (2019) https://doi.org/10.1371/journal.pcbi.1006402

[55] Erdem, C., *et al.*: A scalable, open-source implementation of a large-scale mechanistic model for single cell proliferation and death signaling. Nat Commun **13**(3555), 1–18 (2022) https://doi.org/10.1038/s41467-022-31138-1

[56] Celora, G.L., *et al.*: A DNA-structured mathematical model of cell-cycle progression in cyclic hypoxia. J. Theor. Biol. **545**, 111104 (2022) https://doi.org/10.1016/j.jtbi.2022.111104

[57] Powathil, G.G., Gordon, K.E., Hill, L.A., Chaplain, M.A.J.: Modelling the effects of cell-cycle heterogeneity on the response of a solid tumour to chemotherapy: Biological insights from a hybrid multiscale cellular automaton model. J Theor Biol **308**, 1–19 (2012) https://doi.org/10.1016/j.jtbi.2012.05.015

[58] Shamsi, M., Saghafian, M., Dejam, M., Sanati-Nezhad, A.: Mathematical Modeling of the Function of Warburg Effect in Tumor Microenvironment. Sci. Rep. **8**(8903), 1–13 (2018) https://doi.org/10.1038/s41598-018-27303-6

[59] Jiang, Y., Pjesivac-Grbovic, J., Cantrell, C., Freyer, J.P.: A Multiscale Model for Avascular Tumor Growth. Biophys J **89**(6), 3884–3894 (2005) https://doi.org/10.1529/biophysj.105.060640

[60] Anderson, A.R.A., Rejniak, K.A., Gerlee, P., Quaranta, V.: Modelling of Cancer Growth, Evolution and Invasion: Bridging Scales and Models. Math Model Nat Phenom **2**(3), 1–29 (2007) https://doi.org/10.1051/mmnp:2007001

[61] Zangooei, M.H., Margolis, R., Hoyt, K.: Multiscale computational modeling of cancer growth using features derived from microCT images. Sci. Rep. **11**(18524), 1–17 (2021) https://doi.org/10.1038/s41598-021-97966-1

[62] Owen, M.R., Alarcón, T., Maini, P.K., Byrne, H.M.: Angiogenesis and vascular remodelling in normal and cancerous tissues. J Math Biol **58**(4-5), 689–721 (2009) https://doi.org/10.1007/s00285-008-0213-z

[63] Macklin, P., Edgerton, M.E., Thompson, A.M., Cristini, V.: Patient-calibrated agent-based modelling of ductal carcinoma in situ (DCIS): From microscopic measurements to macroscopic predictions of clinical progression. J. Theor. Biol. **301**, 122–140 (2012) https://doi.org/10.1016/j.jtbi.2012.02.002

[64] Elazab, A., *et al.*: An optimized generic cerebral tumor growth modeling framework by coupling biomechanical and diffusive models with treatment effects. Appl. Soft Comput. **80**, 617–627 (2019) https://doi.org/10.1016/j.asoc.2019.04.034

[65] Ruiz-Martinez, A., *et al.*: Simulations of tumor growth and response to immunotherapy by coupling a spatial agent-based model with a whole-patient quantitative systems pharmacology model. PLoS Comput. Biol. **18**(7), 1010254 (2022) https://doi.org/10.1371/journal.pcbi.1010254

[66] Zhang, S., *et al.*: Integrating single cell sequencing with a spatial quantitative systems pharmacology model spQSP for personalized prediction of triple-negative breast cancer immunotherapy response. ImmunoInformatics **1-2**, 100002 (2021) https://doi.org/10.1016/j.immuno.2021.100002

[67] Nikfar, M., Mi, H., Gong, C., Kimko, H., Popel, A.S.: Quantifying Intratumoral Heterogeneity and Immunoarchitecture Generated In-Silico by a Spatial Quantitative Systems Pharmacology Model. Cancers **15**(10) (2023) https://doi.org/10.3390/cancers15102750

[68] Bergman, D.R., Wang, Y., Trujillo, E., Pearson, A.T., Jackson, T.L.: Dysregulated FGFR3 signaling alters the immune landscape in bladder cancer and

presents therapeutic possibilities in an agent-based model. Front. Immunol. **15**, 1358019 (2024) https://doi.org/10.3389/fimmu.2024.1358019

[69] Kather, J.N., *et al.*: In Silico Modeling of Immunotherapy and Stroma-Targeting Therapies in Human Colorectal Cancer. Cancer Res. **77**(22), 6442–6452 (2017) https://doi.org/10.1158/0008-5472.CAN-17-2006

[70] Simms, K., Bean, N., Koerber, A.: A Mathematical Model of Cell Cycle Progression Applied to the MCF-7 Breast Cancer Cell Line. Bull Math Biol **74**(3), 736–767 (2012) https://doi.org/10.1007/s11538-011-9700-2

[71] Abroudi, A., Samarasinghe, S., Kulasiri, D.: A comprehensive complex systems approach to the study and analysis of mammalian cell cycle control system in the presence of DNA damage stress. J Theor Biol **429**, 204–228 (2017) https://doi.org/10.1016/j.jtbi.2017.06.018

[72] Alkan, O., et al.: Modeling chemotherapy-induced stress to identify rational combination therapies in the DNA damage response pathway. Sci Signaling **11**(540) (2018) https://doi.org/10.1126/scisignal.aat0229

[73] Bae, H., Go, Y.-H., Kwon, T., Sung, B.J., Cha, H.-J.: A Theoretical Model for the Cell Cycle and Drug Induced Cell Cycle Arrest of FUCCI Systems with Cell-to-Cell Variation during Mitosis. Pharm Res **36**(4), 1–13 (2019) https://doi.org/10.1007/s11095-019-2570-2

[74] Basse, B., Ubezio, P.: A Generalised Age- and Phase-Structured Model of Human Tumour Cell Populations Both Unperturbed and Exposed to a Range of Cancer Therapies. Bull Math Biol **69**(5), 1673–1690 (2007) https://doi.org/10.1007/s11538-006-9185-6

[75] Chaffey, G.S., Lloyd, D.J.B., Skeldon, A.C., Kirkby, N.F.: The Effect of the G1 - S transition Checkpoint on an Age Structured Cell Cycle Model. PLoS One **9**(1), 83477 (2014) https://doi.org/10.1371/journal.pone.0083477

[76] Pisu, M., Concas, A., Cao, G.: A novel quantitative model of cell cycle progression based on cyclin-dependent kinases activity and population balances. Comput Biol Chem **55**, 1–13 (2015) https://doi.org/10.1016/j.compbiolchem.2015.01.002

[77] Lonati, L., Barbieri, S., Guardamagna, I., Ottolenghi, A., Baiocco, G.: Radiation-induced cell cycle perturbations: a computational tool validated with flow-cytometry data. Sci Rep **11**(925), 1–14 (2021) https://doi.org/10.1038/s41598-020-79934-3

[78] Falcetta, F., Lupi, M., Colombo, V., Ubezio, P.: Dynamic Rendering of the Heterogeneous Cell Response to Anticancer Treatments. PLoS Comput Biol **9**(10), 1003293 (2013) https://doi.org/10.1371/journal.pcbi.1003293

[79] Tate, S.C., *et al.*: Semi-Mechanistic Pharmacokinetic/Pharmacodynamic Modeling of the Antitumor Activity of LY2835219, a New Cyclin-Dependent Kinase 4/6 Inhibitor, in Mice Bearing Human Tumor Xenografts. Clin Cancer Res **20**(14), 3763–3774 (2014) https://doi.org/10.1158/1078-0432.CCR-13-2846

[80] Ma, C., Almasan, A., Gurkan-Cavusoglu, E.: Computational analysis of 5-fluorouracil anti-tumor activity in colon cancer using a mechanistic pharmacokinetic/pharmacodynamic model. PLoS Comput Biol **18**(11) (2022) https://doi.org/10.1371/journal.pcbi.1010685

[81] Miao, X., Koch, G., Ait-Oudhia, S., Straubinger, R.M., Jusko, W.J.: Pharmacodynamic Modeling of Cell Cycle Effects for Gemcitabine and Trabectedin Combinations in Pancreatic Cancer Cells. Front Pharmacol **7**, 223211 (2016) https://doi.org/10.3389/fphar.2016.00421

[82] Zhu, X., Straubinger, R.M., Jusko, W.J.: Mechanism-based mathematical modeling of combined gemcitabine and birinapant in pancreatic cancer cells. J Pharmacokinet Pharmacodyn **42**(5), 477–496 (2015) https://doi.org/10.1007/s10928-015-9429-x

[83] Jackson, R.C., *et al.*: Modelling of the cancer cell cycle as a tool for rational drug development: A systems pharmacology approach to cyclotherapy. PLoS Comput. Biol. **13**(5), 1005529 (2017) https://doi.org/10.1371/journal.pcbi.1005529

[84] Xie, H., *et al.*: Modeling three-dimensional invasive solid tumor growth in heterogeneous microenvironment under chemotherapy. PLoS One **13**(10), 0206292 (2018) https://doi.org/10.1371/journal.pone.0206292

[85] Norton, K.-A., Wallace, T., Pandey, N.B., Popel, A.S.: An agent-based model of triple-negative breast cancer: the interplay between chemokine receptor CCR5 expression, cancer stem cells, and hypoxia. BMC Syst Biol **11**(1), 1–15 (2017) https://doi.org/10.1186/s12918-017-0445-x

[86] Meacci, L., Primicerio, M.: Interaction between crowding and growth in tumours with stem cells: Conceptual mathematical modelling. Math. Model. Nat. Phenom. **18**, 15 (2023) https://doi.org/10.1051/mmnp/2023011

[87] Bernard, D., *et al.*: A checkpoint-oriented cell cycle simulation model. Cell Cycle **18**(8), 795–808 (2019) https://doi.org/10.1080/15384101.2019.1591125

[88] Gurkan, E., Schupp, J.E., Aziz, M.A., Kinsella, T.J., Loparo, K.A.: Probabilistic Modeling of DNA Mismatch Repair Effects on Cell Cycle Dynamics and Iododeoxyuridine-DNA Incorporation. Cancer Res **67**(22), 10993–11000 (2007) https://doi.org/10.1158/0008-5472.CAN-07-0966

[89] Gurkan-Cavusoglu, E., Schupp, J.E., Kinsella, T.J., Loparo, K.A.: Quantitative analysis of the effects of iododeoxyuridine and ionising radiation treatment on

the cell cycle dynamics of DNA mismatch repair deficient human colorectal cancer cells. IET Syst Biol **7**(4), 114–124 (2013) https://doi.org/10.1049/iet-syb.2012.0050

[90] Kuenzi, B.M., *et al.*: Predicting Drug Response and Synergy Using a Deep Learning Model of Human Cancer Cells. Cancer Cell **38**(5), 672–6846 (2020) https://doi.org/10.1016/j.ccell.2020.09.014

[91] Zhang, H., Chen, Y., Li, F.: Predicting Anticancer Drug Response With Deep Learning Constrained by Signaling Pathways. Front Bioinform **1**, 639349 (2021) https://doi.org/10.3389/fbinf.2021.639349

[92] Gerdes, H., *et al.*: Drug ranking using machine learning systematically predicts the efficacy of anti-cancer drugs. Nat Commun **12**(1850), 1–15 (2021) https://doi.org/10.1038/s41467-021-22170-8

[93] Jeon, J., *et al.*: A systematic approach to identify novel cancer drug targets using machine learning, inhibitor design and high-throughput screening. Genome Med. **6**(7), 1–18 (2014) https://doi.org/10.1186/s13073-014-0057-7

[94] Madhukar, N.S., *et al.*: A Bayesian machine learning approach for drug target identification using diverse data types. Nat. Commun. **10**(5221), 1–14 (2019) https://doi.org/10.1038/s41467-019-12928-6

[95] Ruscone, M., *et al.*: Multiscale model of the different modes of cancer cell invasion. Bioinformatics **39**(6), 374 (2023) https://doi.org/10.1093/bioinformatics/btad374

[96] Wang, Z., Bordas, V., Sagotsky, J., Deisboeck, T.S.: Identifying therapeutic targets in a combined EGFR–TGFβR signalling cascade using a multiscale agent-based cancer model. Math Med Biol **29**(1), 95 (2012) https://doi.org/10.1093/imammb/dqq023

[97] Poltz, R., Naumann, M.: Dynamics of p53 and NF-κB regulation in response to DNA damage and identification of target proteins suitable for therapeutic intervention. BMC Syst Biol **6**(1), 1–19 (2012) https://doi.org/10.1186/1752-0509-6-125

[98] Esteban-Medina, M., Peña-Chilet, M., Loucera, C., Dopazo, J.: Exploring the druggable space around the Fanconi anemia pathway using machine learning and mechanistic models. BMC Bioinf **20** (2019) https://doi.org/10.1186/s12859-019-2969-0

[99] Tzamali, E., Tzedakis, G., Sakkalis, V.: Modeling How Heterogeneity in Cell Cycle Length Affects Cancer Cell Growth Dynamics in Response to Treatment. Front Oncol **10**, 538468 (2020) https://doi.org/10.3389/fonc.2020.01552

[100] Lorz, A., Lorenzi, T., Hochberg, M.E., Clairambault, J., Perthame, B.: Populational adaptive evolution, chemotherapeutic resistance and multiple anti-cancer therapies. ESAIM: M2AN **47**(2), 377–399 (2013) https://doi.org/10.1051/m2an/2012031

[101] Marcu, L., Bezak, E., Olver, I., Doorn, T.: Tumour resistance to cisplatin: a modelling approach. Phys Med Biol **50**(1), 93 (2004) https://doi.org/10.1088/0031-9155/50/1/008

[102] Sun, X., Bao, J., Shao, Y.: Mathematical Modeling of Therapy-induced Cancer Drug Resistance: Connecting Cancer Mechanisms to Population Survival Rates. Sci. Rep. **6**(22498), 1–12 (2016) https://doi.org/10.1038/srep22498

[103] Schmitz, J.E., Kansal, A.R., Torquato, S.: A cellular automaton model of brain tumor treatment and resistance. Journal of Theoretical Medicine **4**(4), 223–239 (2002) https://doi.org/10.1080/1027366031000086674

[104] Frieboes, H.B., *et al.*: Prediction of drug response in breast cancer using integrative experimental/computational modeling. Cancer Res **69**(10), 4484 (2009) https://doi.org/10.1158/0008-5472.CAN-08-3740

[105] Yang, H., Lin, H., Sun, X.: Multiscale modeling of drug resistance in glioblastoma with gene mutations and angiogenesis. Comput Struct Biotechnol J **21:5285-5295.** (2023) https://doi.org/10.1016/j.csbj.2023.10.037

[106] Powathil, G.G., Chaplain, M.A., Swat, M.: Investigating the development of chemotherapeutic drug resistance in cancer: A multiscale computational study (2014). https://doi.org/10.48550/arXiv.1407.0865

[107] Hamis, S., Nithiarasu, P., Powathil, G.G.: What does not kill a tumour may make it stronger: In silico insights into chemotherapeutic drug resistance. J. Theor. Biol. **454**, 253–267 (2018) https://doi.org/10.1016/j.jtbi.2018.06.014

[108] Sun, X., *et al.*: Mathematical modeling reveals a critical role for cyclin D1 dynamics in phenotype switching during glioma differentiation. FEBS Lett **589**(18), 2304–2311 (2015) https://doi.org/10.1016/j.febslet.2015.07.014

[109] Komarova, N.: Stochastic modeling of drug resistance in cancer. J Theor Biol **239**(3), 351–366 (2006) https://doi.org/10.1016/j.jtbi.2005.08.003

[110] Sun, X., *et al.*: Stochastic modeling suggests that noise reduces differentiation efficiency by inducing a heterogeneous drug response in glioma differentiation therapy. BMC Syst Biol **10**(1), 1–13 (2016) https://doi.org/10.1186/s12918-016-0316-x

[111] Chen, C., et al.: Mathematical models of the transitions between endocrine therapy responsive and resistant states in breast cancer. J R Soc Interface **11**(96)

(2014) https://doi.org/10.1098/rsif.2014.0206

[112] Hirata, Y., Tanaka, G., Bruchovsky, N., Aihara, K.: Mathematically modelling and controlling prostate cancer under intermittent hormone therapy. Asian J Androl **14**(2), 270 (2012) https://doi.org/10.1038/aja.2011.155

[113] Jain, H.V., Clinton, S.K., Bhinder, A., Friedman, A.: Mathematical modeling of prostate cancer progression in response to androgen ablation therapy. Proc Natl Acad Sci U.S.A **108**(49), 19701–19706 (2011) https://doi.org/10.1073/pnas.1115750108

[114] Billy, F., Clairambaultt, J., Fercoq, O., Gaubertt, S., Lepoutre, T., Ouillon, T., Saito, S.: Synchronisation and control of proliferation in cycling cell population models with age structure. Math Comput Simul **96**, 66–94 (2014) https://doi.org/10.1016/j.matcom.2012.03.005

[115] Hesse, J., Martinelli, J., Aboumanify, O., Ballesta, A., Relógio, A.: A mathematical model of the circadian clock and drug pharmacology to optimize irinotecan administration timing in colorectal cancer. Comput Struct Biotechnol J **19**, 5170–5183 (2021) https://doi.org/10.1016/j.csbj.2021.08.051

[116] Panetta, J.C., Fister, K.R.: Optimal control applied to cell-cycle-specific cancer chemotherapy. SIAM Journal on Applied Mathematics **60**(3), 1059–1072 (2000) https://doi.org/10.1137/S0036139998338509

[117] Pillis, L.G., *et al.*: Chemotherapy for tumors: An analysis of the dynamics and a study of quadratic and linear optimal controls. Math Biosci **209**(1), 292–315 (2007) https://doi.org/10.1016/j.mbs.2006.05.003

[118] Dua, P., Dua, V., Pistikopoulos, E.N.: Optimal delivery of chemotherapeutic agents in cancer. Comput Chem Eng **32**(1), 99–107 (2008) https://doi.org/10.1016/j.compchemeng.2007.07.001

[119] Alam, M.S., *et al.*: Multi-objective multi-drug scheduling schemes for cell cycle specific cancer treatment. Comput Chem Eng **58**, 14–32 (2013) https://doi.org/10.1016/j.compchemeng.2013.05.021

[120] Kim, K.S., Cho, G., Jung, I.H.: Optimal Treatment Strategy for a Tumor Model under Immune Suppression. Comput Math Methods Med **2014** (2014) https://doi.org/10.1155/2014/206287

[121] Leder, K., *et al.*: Mathematical Modeling of PDGF-Driven Glioblastoma Reveals Optimized Radiation Dosing Schedules. Cell **156**(3), 603–616 (2014) https://doi.org/10.1016/j.cell.2013.12.029

[122] Clairambault, J., Fercoq, O.: Physiologically Structured Cell Population Dynamic Models with Applications to Combined Drug Delivery Optimisation

in Oncology. Math. Model. Nat. Phenom. **11**(6), 45–70 (2016) https://doi.org/10.1051/mmnp/201611604

[123] Lai, X., Friedman, A.: Mathematical modeling in scheduling cancer treatment with combination of VEGF inhibitor and chemotherapy drugs. J Theor Biol **462**, 490–498 (2019) https://doi.org/10.1016/j.jtbi.2018.11.018

[124] Kuznetsov, M., Kolobov, A.: Optimization of antitumor radiotherapy fractionation via mathematical modeling with account of 4 R's of radiobiology. J. Theor. Biol. **558**, 111371 (2023) https://doi.org/10.1016/j.jtbi.2022.111371

[125] Powathil, G.G., Adamson, D.J.A., Chaplain, M.A.J.: Towards Predicting the Response of a Solid Tumour to Chemotherapy and Radiotherapy Treatments: Clinical Insights from a Computational Model. PLoS Comput Biol **9**(7), 1003120 (2013) https://doi.org/10.1371/journal.pcbi.1003120
